# Bioinformatics analysis reveals the association of bile acid metabolism-related genes with sepsis

**DOI:** 10.1371/journal.pone.0352431

**Published:** 2026-07-10

**Authors:** Juan Xie, Yu Ling, Yunyu Sun, Yun Yao, Mingshun Zhang, Xiaoyu Zhou

**Affiliations:** 1 Department of Immunology, Nanjing Medical University, Nanjing, China; 2 Department of Transfusion, The First Affiliated Hospital of Nanjing Medical University, Nanjing, China; 3 Clinical Laboratory, Women’s Hospital of Nanjing Medical University, Nanjing Women and Children’s Healthcare Hospital, Nanjing, China; The First Hospital of Jilin University, CHINA

## Abstract

**Background:**

Sepsis, is a life-threatening syndrome triggered by infection. Bile acid metabolism may be involved in the pathogenesis of sepsis, the underlying association has not yet been elucidated. Thus, we aimed to screen for bile acid metabolism-related biomarkers of sepsis and discover the potential association.

**Methods:**

Sepsis-related datasets were downloaded from the Gene Expression Omnibus (GEO) database. We identified differentially expressed genes (DEGs) and bile acid metabolism-related differentially expressed genes (BAMRDEGs), then identified hub genes using protein–protein interaction (PPI) network analysis and CytoHubba algorithm. After GO/KEGG enrichment analysis, a sepsis risk prediction model based on key genes was subsequently constructed using support vector machine recursive feature elimination (SVM-RFE) machine learning and least absolute shrinkage and selection operator (LASSO) regression. CIBERSORTx analysis was performed to assess immune cell infiltration and its association with key genes. Finally, the transcriptional levels of key genes in sepsis samples were detected by Quantitative Real-time PCR (qRT-PCR).

**Results:**

9785 DEGs were identified, including 5138 upregulated and 4647 downregulated genes. Additionally, 25 hub genes were identified. Gene enrichment analysis indicated that the hub genes participate in multiple biological pathways. Key genes (*ABCC2*, *PECR*, *EPHX2*, *PEX2*, and *AGXT*) exert central roles in the development of sepsis, indicating the involvement of bile acid metabolism. Significant correlations existed between the expression of key BAMRDEGs and the levels of different immune cell types. qRT-PCR suggested significant up-regulation on *ABCC2* and *AGXT* in sepsis samples versus controls.

**Conclusion:**

This study revealed novel insights into the correlation between sepsis and bile acid metabolism, and identified 5 key genes involved in the development of sepsis, providing molecular targets and novel strategies for the diagnosis and treatment of sepsis.

## 1. Introduction

Sepsis is a fatal clinical syndrome induced by dysregulation of the host response to infection. A global burden of disease study estimated that 48.9 million incident cases of sepsis occurred worldwide between 1990 and 2017, of which 11 million resulted in death, representing approximately 19.7% of all global deaths [1]. Sepsis has become a challenging global health issue because of its high mortality and disability rates. With disease progression and sustained systemic inflammation, severe sepsis typically leads to multiple organ dysfunction syndrome (MODS), organ hypoperfusion, or hypotension, which poses an enormous challenge for treating sepsis. Currently, the diagnostic and treatment methods for sepsis mainly depend on laboratory tests and supportive therapy but lack effective and specific diagnostic markers. Thus, further exploration of more effective biomarkers for early diagnosis and treatment of sepsis is imperative.

The pathogenesis of sepsis is complex, implicating multiple factors such as immune response imbalance, cellular energy metabolism disorder, and endogenous toxin release [2]. Recently, the involvement of bile acid metabolism in the development of sepsis has received increasing attention. Cholestasis is one of the earlier events in sepsis pathology [3]. Bile acid metabolism is significantly dysregulated in patients with sepsis, with elevated bile acid and decreased metabolite levels [4]. During sepsis, upregulated inflammatory mediators inhibit the expression of bile acid transporters sodium-dependent taurocholate co-transporting polypeptide (NTCP) and bile salt export pump (BSEP), and resulting in impaired bile acid metabolism, thus causing cholestasis [5]. Excessive levels of bile acids activate the NOD-like receptor thermal protein domain-associated protein 3 (NLRP3) in liver which promotes the release of proinflammatory factors, in turn exacerbating either liver localized or systemic inflammatory response [6]. These findings could highlight the importance of exploring the relationship between bile acid metabolism and sepsis. Hence, identification of novel biomarkers related to bile acid metabolism in sepsis is important for early diagnosis and disease progression.

In this study, we utilized multiple bioinformatic approaches, including batch effects removal, differential expression analysis, PPI network analysis, enrichment analysis, and machine learning model construction. The main advantage of these techniques is the comprehensive utilization of different datasets for integrated analysis, which improves the accuracy and reliability of the research results. We used bioinformatics analysis to explore the expression profiles of bile acid metabolism-related genes in sepsis to provide novel insight toward the underlying causative mechanisms and diagnostic approaches for sepsis.

## 2. Materials and methods

### 2.1 Data sources and preprocessing

The sepsis databases (GSE28750 [7] and GSE95233 [8] and GSE13904 [9]) used in this study were fetched from GEO database [10] using the “GEOquery” R package [11]. Dataset GSE28750 contains 10 sepsis and 20 normal samples. Dataset GSE95233 contains 102 sepsis and 22 normal samples. Dataset GSE13904 contains 154 sepsis and 18 normal samples, and 52 and 102 patients with sepsis and septic shock, respectively. The samples from these datasets were blood tissues from Homo sapiens and were based on the GPL17077 platform. Detailed information on datasets was presented in Table 1.

**Table 1:** GEO microarray chip information.

	GSE28750	GSE95233	GSE13904
Platform	GPL570	GPL570	GPL570
Species	Homo sapiens	Homo sapiens	Homo sapiens
Tissue	Blood	Blood	Blood
Samples in Sepsis group	10	102	154
Samples in Control group	20	22	18
Reference	PMID: 21682927	PMID: 28341250	PMID: 19325468

To ensure the comparability among different datasets and the reliability of data integration, the probe numbers were uniformly converted into gene symbols based on corresponding platform annotation files. To minimize technology platform differences and non-biological noise, the batch effects correction were performed on the GSE28750 and GSE95233 datasets using the ComBat function in the “sva” R package [12], the combined datasets was obtained, involving 112 sepsis and 42 normal samples. To reduce technical variations among samples, dataset GSE13904 as well as the combined datasets were standardized and normalized using the normalizeBetweenArrays function in the “limma” R package [13].The combined datasets was used as the test dataset, and dataset GSE13904 was used as the validation dataset. This approach has been the standard method for multi-chip integrated analysis, improving the consistency and reliability of cross-dataset analysis. To validate the effect of the batch removal, principal component analysis (PCA) was conducted on the expression matrix before and after removing the batch. PCA is a data dimensionality reduction method that converts feature vectors extracted from high-dimensional data into low-dimensional data and presents these features in two-dimensional or three-dimensional graphs [14].

### 2.2 Screening of DEGs related to sepsis-associated bile acid metabolism

First, we confirmed that the samples in the combined datasets were divided into sepsis and normal groups, used the “limma” R package to conduct differential gene analysis, applying the thresholds of |logFC| > 0 and p value <0.05. Genes with logFC > 0 and logFC < 0 were considered upregulated and downregulated DEGs, respectively. The results were visualized in a volcano plot constructed using the “ggplot2” R package. Limma algorithm is widely used in the screening of disease-related DEGs as it has mature linear model framework and robust statistical performance in high-throughput microarray and transcriptome data analysis, and yields high sensitivity and specificity with a limited sample size [13].

Additionally, bile acid metabolism-related genes were incorporated from the Gene Cards (GC) database [15] and the published literature in PubMed website by searching keyword “bile acid metabolism” [16,17]. After eliminating duplicates, 200 BAMRGs were identified. Detailed information was provided in S1 Table. To identify BAMRDEGs associated with sepsis, we extracted the intersecting genes between DEGs and BAMRGs. A Venn diagram was used to visualize the results. Subsequently, the top 20 BAMRDEGs were visualized in a heatmap generated using the “pheatmap” R package.

### 2.3 PPI network and hub gene screening

STRING [18] database and the cytoHubba [19] plug-in of Cytoscape [20] software are internationally validated tools for analyzing protein interactions and network topologies, offer advantages such as wide interactive coverage, intuitive visualization of results, and strong reproducibility. The combined application of the two methods is particularly suitable for identifying key functional genes and core regulatory networks in complex biological systems. In this study, PPI network was constructed based on STRING database, with a minimum interaction coefficient >0.400. Tightly linked localized regions in PPI network might denote molecular complexes with distinctive biological functions. Genes in the PPI network that interacted with other genes were selected for subsequent analyses.

In addition, five algorithms from cytoHubba were applied to count the scores of the BAMRDEGs: maximal clique centrality (MCC), degree, maximum neighborhood component (MNC), edge percolated component (EPC), and closeness [21]. The top 30 BAMRDEGs were selected according to their scores. Lastly, the hub genes of the BAMRDEGs were obtained by considering the intersecting genes generated by five different algorithms, and the results were illustrated using a Venn plot.

### 2.4 Differential expression verification and Receiver Operating Characteristic (ROC) curve analysis

To probe the expression differences in hub genes between the sepsis and normal groups, a group comparison map was constructed based on the expression levels of hub gene. The ROC curves of the hub genes were plotted and the area under the curve (AUC) values were calculated using the “pROC” R package [22] to assess the diagnostic performance of hub gene expression for sepsis. The AUC is typically in the range 0.5−1. The higher the AUC value (closer to 1) is, the better the diagnostic accuracy. The diagnostic accuracy is low, medium, and high when the AUC is between 0.5–0.7, 0.7–0.9, and > 0.9, respectively.

### 2.5 Gene Ontology (GO) function and Kyoto Encyclopedia of Genes and Genomes (KEGG) enrichment analysis

GO [23] and KEGG [24] analysis systems are international standard frameworks for elucidating gene functions and signaling pathways, and have been extensively used for studying the disease mechanisms. The statistical models with long-term verifications could be used to systematically reveal the roles of DEGs in biological processes (BP), molecular functions (MF), and cellular components (CC). In this study, the “clusterProfiler” R package [25] was used for GO and KEGG enrichment analysis to explore the potential biological functions of hub genes, applying the entry screening criteria of adjusted p < 0.05 and FDR value (q value) <0.25. The Benjamini-Hochberg (BH) p-correction method was used.

### 2.6 Gene Set Enrichment Analysis (GSEA)

GSEA [26] is widely used in the functional trend analysis of multiple diseases owning to its statistical robustness and sensitive detection ability for the overall expression trend of the gene set. To reveal potential changes in signaling pathways, this study utilized GSEA to evaluate the enrichment characteristics at the gene set level to complement the results of single-gene difference analysis. The “clusterProfiler” R package was used to perform GSEA. The parameters were as follows: the seed number was 2022, the computations number was 1000, the maximum and minimum number of genes contained in each gene set are 500 and 10, respectively. The gene set “c2.all.v2024.1. Hs.symbols.gmt” was acquired from the Molecular Signatures Database [27] for enrichment analysis, applying the screening criteria of adjusted p < 0.05 and FDR value (q value) <0.25. BH was used as the adjusted p correction method.

### 2.7 Construction and validation of sepsis diagnostic model

In order to construct a sepsis diagnostic model, a combination of three classical machine learning methods was applied in this study (logistic regression, SVM-RFE, and LASSO regression). These algorithms could balance model stability and biological interpretability, and have been extensively validated in the analysis of small sample high-dimensional data. Logistic regression reveals the linear correlation between the hub genes and sepsis occurrence, adopting the screening criteria of p-value <0.05. SVM-RFE relies on the feature selection algorithm of support vector machine (SVM) to select the most important features by recursively eliminating those that contribute the least to the classification. To identify potential biomarkers among the hub genes included in the logistic regression model, the SVM-RFE algorithm was performed using the “e1071” R package. LASSO regression analysis is routinely applied to construct prognostic models, and a penalty term (lambda × absolute value of the slope) is added based on linear regression to improve the generalizability of the model and prevent overfitting. Based on the hub genes contained in the SVM-RFE algorithm, the sepsis diagnostic model and key genes were developed using LASSO regression analysis that was conducted using the “glmnet” R package [28], with set seed (500) and family = “binomial” as parameters, and the results were presented using a diagnostic model and variable trajectory diagrams. The LASSO risk score was calculated according to the risk coefficient of LASSO regression analysis, using the following formula:


$$\begin{array}{c} \text{risk}Score=\sum_{i}Coefficient\left({gene}_{i}\right)*mRNAExpression({gene}_{i}) \end{array}$$

To evaluate the diagnostic performance of risk score for the occurrence of sepsis, the ROC curves of the combined datasets and dataset GSE13904 were plotted, and the AUC values were calculated using the “pROC” R package [22]. The nomogram was plotted to display the interrelationships of key genes using the “rms” R package. A nomogram visually expresses the functional relationships between several independent variables in cartesian coordinate system using a set of disjoint line segments [29]. To assess the accuracy and discrimination of the sepsis diagnostic model, the calibration curves were plotted based on the risk scores of the combined datasets and dataset GSE13904, using the“ggDCA”R package. Decision curve analysis (DCA) is a potent tool used to evaluate molecular markers, clinical prediction models, and diagnostic tests [30].

### 2.8 Friends analysis

The semantic comparison of GO [23] annotation is an approach for computing similarity between genes and genomes, which is particularly useful for supporting many bioinformatic analysis methods. The “GOSemSim” R package [31] was applied to perform functional similarity (friend) analysis of key genes.

### 2.9 Correlation analysis and Gene Set Enrichment Analysis (GSEA)

Sepsis samples from the combined datasets were assigned into high- and low-risk groups based on the median LASSO risk score of the sepsis diagnostic model. To investigate differences in the expression of key genes between the two groups, a comparison chart of gene expression levels was generated. To further probe the correlations among the key genes, we performed a correlation analysis of the expression levels of key genes using the Spearman algorithm, drew a corresponding heat map using the “pheatmap” R package, and screened the key genes with the strongest correlation to draw a correlation scatter plot using the “ggplot2” R package. An absolute value of the correlation coefficient less than 0.3, between 0.3 and 0.5, between 0.5 and 0.8, and greater than 0.8 represent no, weak, moderate and strong correlations, respectively. DEGs analysis was performed using the “limma” R package, and the results were presented as a volcano map constructed using the “ggplot2” R package. The top 20 DEGs were identified in descending order of |logFC| and the results were visualized using a heat map. The DEGs were sorted base on their logFC values, and GSEA was performed on all genes using the “clusterProfiler” R package.

### 2.10 Construction of regulatory network

Transcription factors (TFs) regulate gene expression through interacting with key genes at the post-transcriptional stage. We retrieved TFs from the ChIPBase database [32], analyzed their regulatory effects on key genes. Furthermore, miRNAs exert vital roles in biological development and evolution by regulating the expression of target genes. To identify the relationships between key genes and miRNAs, the key genes-related miRNAs were retrieved from the StarBase v3.0 database [33]. RNA-binding proteins (RBPs) [34] exert pivotal roles in gene regulation, including RNA synthesis, modification, selective splicing, translation, and transport. The target RBPs of key genes were predicted using StarBase v3.0 database [33]. To identify the interactions between hub genes and drugs, the drug targets of hub genes were predicted using Comparative Toxicogenomics Database (CTD) [35]. Lastly, the regulatory networks of mRNA-TFs, mRNA-miRNA, mRNA-RBP and mRNA-drug were visually presented using Cytoscape [20].

### 2.11 Prediction of protein domain

Proteins are crucial for basic life functions, and understanding their structures could improve our understanding of their functions. The AlphaFoldDB database [36] contains approximately 350, 000 protein structures from humans and 20 model organisms universally applied in biological research, such as Escherichia coli, mice, zebrafish and fruit flies. The protein structures of key genes were visually predicted using AlphaFoldDB database and the predicted local distance difference test (pLDDT) ranged from 0 to 100. The pLDDT less than 50, between 50 and 70, between 70 and 90, and greater than 90 indicate low, medium, high, very high confidence, respectively.

### 2.12 Immune infiltration analysis

To evaluate the infiltration characteristics of immune cells in sepsis, CIBERSORT algorithm was utilized. The CIBERSORT algorithm is an immune deconvolution analysis method based on linear support vector regression and could accurately estimate immune cell composition from mixed tissue transcriptome data. The robustness of this algorithm in blood sample analysis has been widely verified. Its original method is based on the LM22 leukocyte signature matrix, primarily used to estimate the relative abundance of 22 types of human immune cell subsets in complex mixed samples, the establishment of methodology itself includes the modeling and validation of the expression characteristics related to leukocytes [37]. Multiple bioinformatics studies based on sepsis peripheral blood or whole blood transcriptomes have employed CIBERSORT to assess immune cell composition and its correlation with key genes, using it as an auxiliary analytical tool to reveal disease-related immune characteristics [38–40]. In this study, combined with the characteristic gene matrix of immune cells, the CIBERSORT algorithm was used to filter out the data with enrichment score of immune cells larger than zero, and the results were presented using a proportion bar chart. Furthermore, the correlations among immune cells as well as the correlations between key genes and immune cells were calculated using Spearman algorithm.

### 2.13 Validation in sepsis patients

To further validate the expression changes of key genes in sepsis, we enrolled 20 patients with sepsis from the intensive care units (ICUs) and 20 healthy controls from the examination center in our hospital between January 2025 and March 2025. All the subjects were over 18 years old. Inclusion criteria for sepsis patients: all patients met the 2016 International Consensus Diagnostic Criteria for sepsis [41], with complete medical records. Patients were excluded if they died within 24 h of ICU admission; were pregnant or nursing women; had autoimmune diseases or hematological diseases.

Whole blood samples were collected from sepsis patients and healthy controls. RNA was extracted from the whole blood samples with RNA extraction kit (R1220; solarbio) in accordance with the manufacturer’s instructions. cDNA was synthesized using TransScript® All-in-One First-Strand cDNA Synthesis SuperMix (AT341; transgen) for qRT-PCR. The qRT-PCR was performed with a fluorescent quantitative PCR instrument (VII7; Thermo). The relative mRNA expression levels of key genes were calculated with the 2^–△△Ct^ method.

This study was strictly performed referring to the Declaration of Helsinki and approved by the First Affiliated Hospital Committee of Nanjing Medical University (ethics number: 2025-SR-346). Based on the confidentiality and low risk of the study, we applied for an exemption from informed consent and verbally informed each subject of the study purpose, method and risks.

### 2.14 Statistical analysis

R software v 4.3.3 was applied to conduct bioinformatic analysis. The independent Student’s *t*-test and Mann-Whitney U test (Wilcoxon rank-sum test) were applied to compare differences between two groups with normal and non-normal distributions, respectively. The Kruskal-Wallis test was employed for multiple-groups comparison, while Spearman’s correlation analysis was used to assess correlations. A p-value <0.05 was considered statistically significant.

## 3. Results

### 3.1 Technology roadmap (Fig 1)

**Fig 1:**
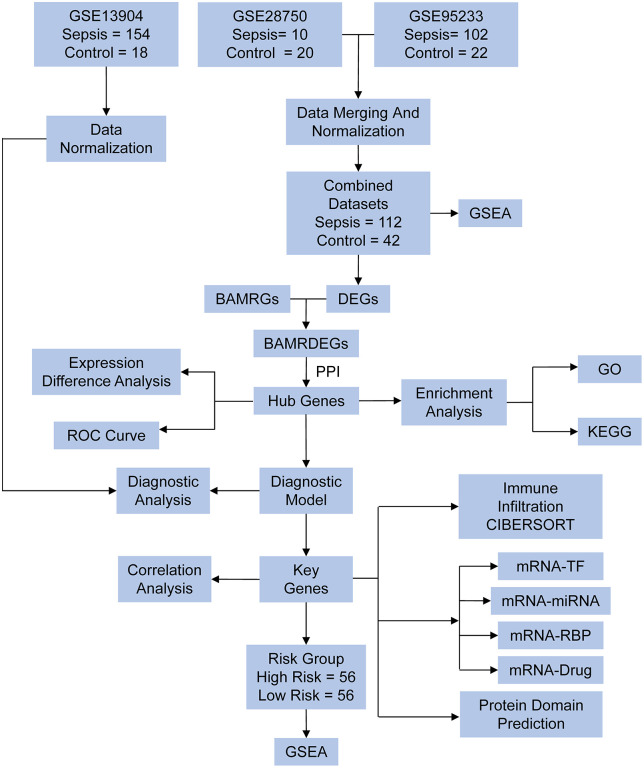
Flow chart for the comprehensive analysis of BAMRDEGs.

### 3.2 Standardization of sepsis datasets

**Fig 2:**
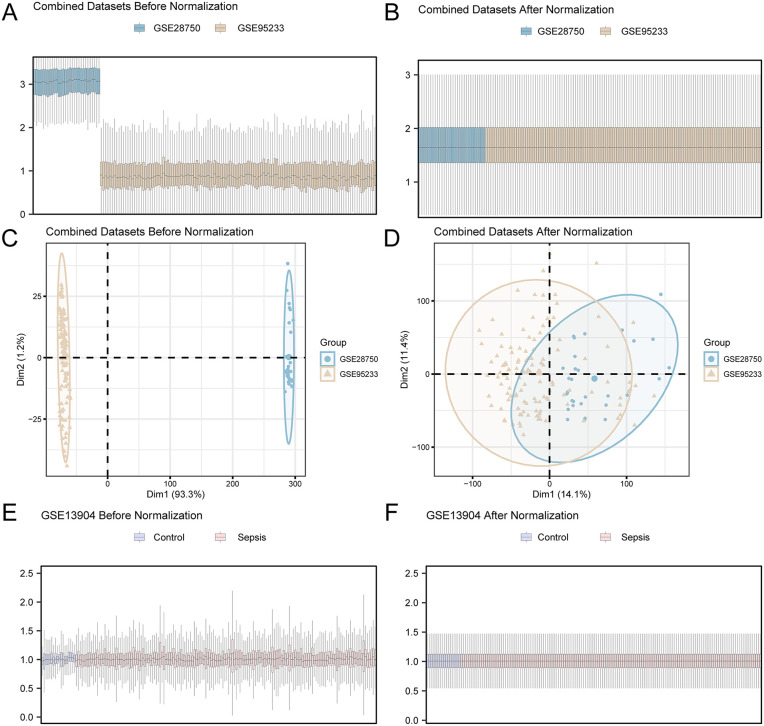
Batch effects removal of sepsis datasets. **(A)** Boxplot distribution of the combined datasets before batch removal; **(B)** Boxplot distribution of the combined datasets after batch removal; **(C)** PCA plot of the combined datasets before batch removal; **(D)** PCA plot of the combined datasets after batch removal; **(E)** Boxplot distribution of dataset GSE13904 before normalization; **(F)** Boxplot distribution of dataset GSE13904 after normalization. The pink denotes the sepsis group, the purple denotes the control group, the yellow denotes dataset GSE95233, and the blue denotes the dataset GSE28750.

After processing, 112 septic and 42 normal samples from the combined datasets were obtained, and the data distributions were plotted before and after removing the batch effect (Fig 2A and 2B). A PCA plot was applied to compare the low-dimensional feature distribution of the combined datasets before and after batch-effect removal (Fig 2C and 2D). The results indicated that the batch effect of the combined datasets was largely eliminated. A distribution boxplot was used to compare the expression levels of dataset GSE13904 before and after normalization (Fig 2E and 2F).

### 3.3 Screening for sepsis-related BAMRDEGs

**Fig 3:**
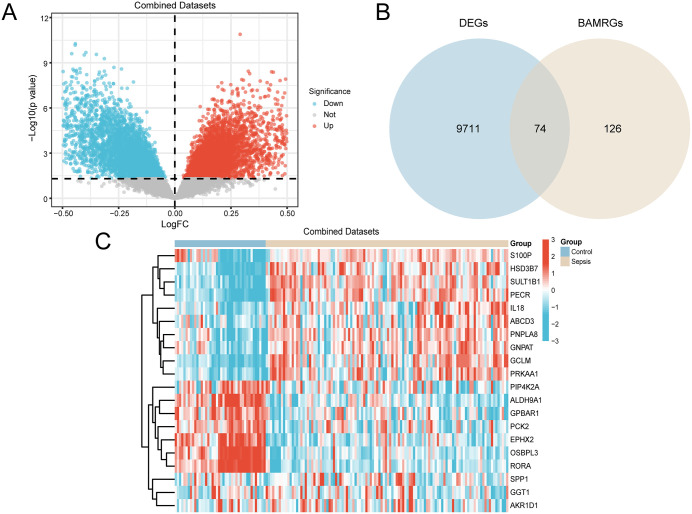
Differential gene expression analysis. **(A)** Volcano plot of DEGs; **(B)** Venn plot of overlap and distinct genes between DEGs and BAMRGs; **(C)** Heat map of the top 20 BAMRDEGs. In the heat map, the yellow represents the sepsis group and the blue represents the control group, the red represents high expression and the blue represents low expression.

A volcano plot of the samples from the combined datasets revealed differences in the distribution of 9785 genes between the two groups, among which 5138 were upregulated and 4647 were downregulated (Fig 3A). We analyzed the intersection of the DEGs and 200 BAMRGs, a Venn plot was generated that identified 74 BAMRDEGs (S1 Table and Fig 3B). Furthermore, the top 20 BAMRDEGs between the sepsis and normal groups in the combined datasets were displayed using a heatmap (Fig 3C).

### 3.4 Construction of PPI network and screening of hub genes

**Fig 4:**
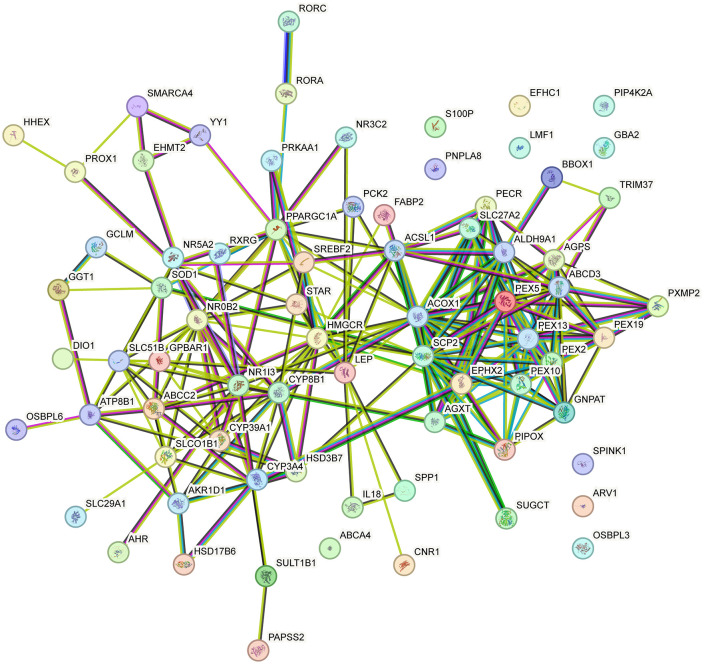
PPI network analysis.

A PPI network of 74 BAMRDEGs was constructed to analyze protein-protein interactions using STRING database (Fig 4). The results showed that 50 BAMRDEGs were interrelated, and the detailed information was shown in S3 Table.

**Fig 5:**
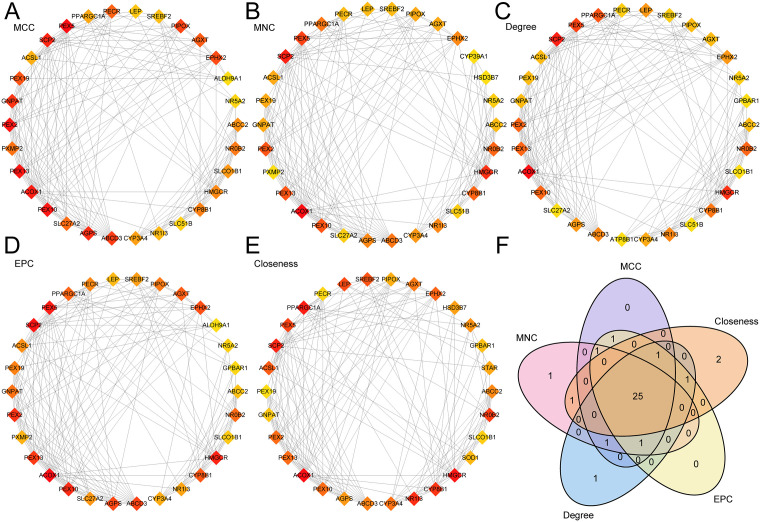
PPI networks and hub genes analysis. **(A-E)** PPI Networks of top 30 BAMRDEGs, including MCC (A), MNC (B), degree (C), EPC (D) and closeness (E); **(F)** Venn plot of Top 30 BAMRDEGs.

The scores of 50 BAMRDEGs were calculated by the five algorithms of cytohubba plug-in, and BAMRDEG were ranked in sequence according to the scores. The PPT networks were plotted based on the top 30 BAMRDEGs among the five algorithms as follows: MCC (Fig 5A), MNC (Fig 5B), degree (Fig 5C), EPC (Fig 5D) and closeness (Fig 5E). Circles ranging from red to yellow denote the scores from high to low. Bile acid metabolism-related hub genes in sepsis were identified by extracting the intersecting genes calculated using the five algorithms (Fig 5F). These 25 hub genes included *PEX5*, *PEX10*, *PEX13*, *PEX2*, *ACOX1*, *SCP2*, *AGPS*, *GNPAT*, *ABCD3*, *PIPOX*, *EPHX2*, *PEX19*, *AGXT*, *PECR*, *NR0B2*, *CYP8B1*, *HMGCR*, *ABCC2*, *CYP3A4*, *PPARGC1A*, *ACSL1*, *NR1I3*, *SREBF2*, *LEP* and *NR5A2.*

### 3.5 Differential expression verification and ROC curve analysis

**Fig 6:**
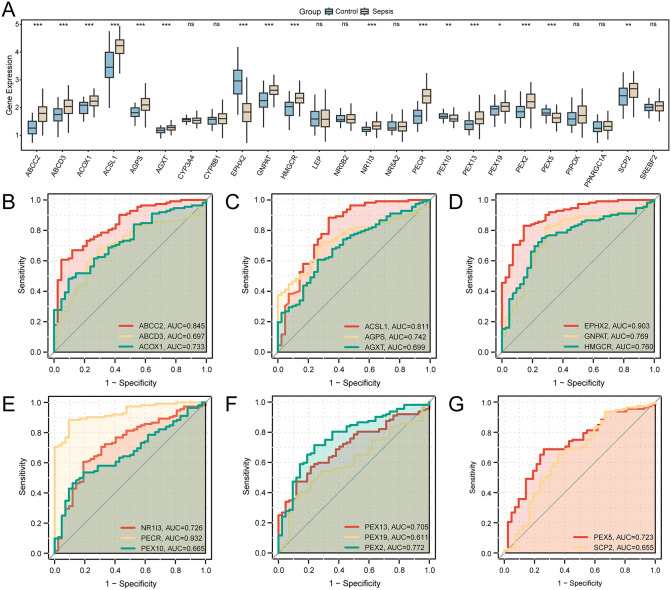
Differential expression validation and ROC curve analysis. **(A)** Group comparison plots of hub genes between sepsis and control groups in the combined datasets; **(B-G)** ROC curves of hub genes in the combined datasets: *ABCC2*, *ABCD3*, *ACOX1* (B); *ACSL1*, *AGPS*, *AGXT* (C); *EPHX2*, *GNPAT*, *HMGCR* (D); *NR1I3*, *PECR*, *PEX10* (E); *PEX13, PEX19, PEX2* (F) *PEX5*, and *SCP2* (G). Blue represents the control group and yellow represents the sepsis group.

When analyzing the expression levels of hub genes between the sepsis and normal groups, we found several statistically significant differences. The difference in *PEX19* expression between the two groups was statistically significant (p value < 0.05). The difference in *PEX10* and *SCP2* expression between the two groups were highly statistically significant (p value < 0.01). The difference in 14 hub genes expression between the two groups were extremely statistically significant (p value < 0.001), namely: *PEX5、PEX13、PEX2、ACOX1、AGPS、GNPAT、ABCD3、EPHX2、AGXT、PECR、HMGCR、ABCC2、ACSL1* and *NR1I3* (Fig 6A).

Subsequently, ROC curves were plotted according to the expression levels of the hub genes using “pROC” R package. The results revealed that the 5 hub genes showed a low ability to distinguish between sepsis and normal groups (0.5 < AUC < 0.7), namely: *ABCD3*, *AGXT*, *PEX10*, *PEX19* and *SCP2*; the 10 hub genes showed a certain ability to distinguish between the two groups (0.7 < AUC < 0.9), namely: *ABCC2*, *ACOX1*, *AGPS*, *ACSL1*, *GNPAT*, *HMGCR*, *NR1I3*, *PEX13*, *PEX2* and *PEX5*; *PECR* and *EPHX2* showed high ability to distinguish between the two groups (AUC > 0.9, Fig 6B–6G).

### 3.6 Gene ontology (GO) and Kyoto Encyclopedia of Genes and Genomes (KEGG) pathway enrichment analysis

**Fig 7:**
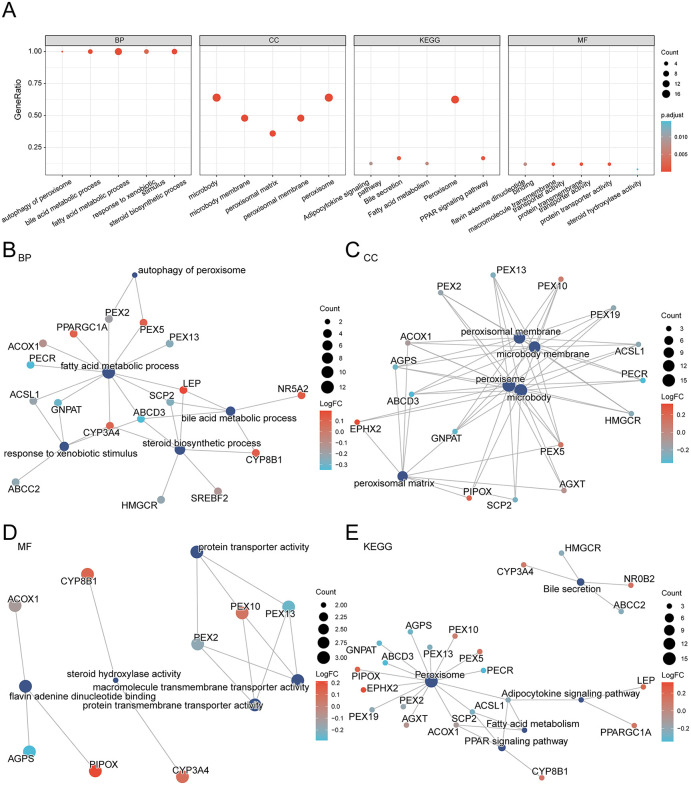
GO and KEGG enrichment analysis. **(A)** Bubble plot of GO and KEGG pathway enrichment analysis of hub genes. The abscissa is GO terms and KEGG terms; **(B-E)** Network diagrams of GO and KEGG pathway enrichment analysis of hub genes: BP (B), CC (C), MF (D) and KEGG (E). Dark blue nodes represent entries, and lines represent the relationship between entries and molecules. In molecular nodes, red represents up-regulation, and blue represents down-regulation. In the bubble plot, the bubble size represents the number of genes and the color represents the size of the adj. p-value. The redder the color is, the smaller the adj. p-value, and the bluer the color is, the larger the adj. p-value.

GO analysis revealed that the 25 hub genes were mainly enriched in fatty acid metabolic processes, bile acid metabolic processes, peroxisome autophagy, response to xenobiotic stimuli, steroid biosynthetic processes, and other BP: peroxisome, microbody, peroxisomal membrane, microbody membrane, peroxisomal matrix, and other CC; protein transmembrane transporter activity, macromolecule transmembrane transporter activity, protein transporter flavin adenine dinucleotide binding, steroid hydroxylase activity, and MF. KEGG pathway enrichment analysis revealed that the 25 hub genes were mainly enriched in the peroxisome, peroxisome proliferator-activated receptor (PPAR) signaling pathway, bile secretion, fatty acid metabolism, adipocytokine signaling pathway. The results were visualized using a bubble plot (Fig 7A). Network diagrams of BP, CC, MF, and biological pathways were plotted (Fig 7B–7E). Detailed information was shown in Table 2. The lines indicate the corresponding molecules and annotations of the corresponding entries. The larger the node, the more molecules are contained in the element.

**Table 2:** Result of GO and KEGG enrichment analysis for BAMRDEGs.

Ontology	ID	Description	Gene Ratio	BgRatio	p value	p.adjust	q value
BP	GO:0006631	fatty acid metabolic process	12/25	401/18870	2.92E-14	1.22E-11	6.82E-12
BP	GO:0008206	bile acid metabolic process	5/25	50/18870	5.43E-09	2.06E-07	1.15E-07
BP	GO:0030242	autophagy of peroxisome	2/25	10/18870	7.53E-05	0.001030043	0.000577229
BP	GO:0009410	response to xenobiotic stimulus	5/25	434/18870	0.000228437	0.002574543	0.001442758
BP	GO:0006694	steroid biosynthetic process	7/25	184/18870	3.10E-09	1.29E-07	7.21E-08
CC	GO:0005777	peroxisome	16/25	143/19886	4.16E-29	6.45E-28	5.48E-28
CC	GO:0042579	microbody	16/25	143/19886	4.16E-29	6.45E-28	5.48E-28
CC	GO:0005778	peroxisomal membrane	12/25	65/19886	2.55E-24	1.98E-23	1.68E-23
CC	GO:0031903	microbody membrane	12/25	65/19886	2.55E-24	1.98E-23	1.68E-23
CC	GO:0005782	peroxisomal matrix	9/25	51/19886	4.51E-18	2.33E-17	1.98E-17
MF	GO:0008320	protein transmembrane transporter activity	3/25	27/18496	6.25E-06	0.000685285	0.00034403
MF	GO:0022884	macromolecule transmembrane transporter activity	3/25	32/18496	1.05E-05	0.000685285	0.00034403
MF	GO:0140318	protein transporter activity	3/25	39/18496	1.93E-05	0.000836537	0.000419962
MF	GO:0050660	flavin adenine dinucleotide binding	3/25	88/18496	0.00022189	0.007211416	0.003620306
MF	GO:0008395	steroid hydroxylase activity	2/25	26/18496	0.000558809	0.014529036	0.007293929
KEGG	hsa04146	Peroxisome	15/24	83/8545	2.08E-25	1.08E-23	8.33E-24
KEGG	hsa03320	PPAR signaling pathway	4/24	76/8545	5.37E-05	0.001394953	0.001073041
KEGG	hsa04976	Bile secretion	4/24	90/8545	0.000104082	0.001804085	0.001387758
KEGG	hsa01212	Fatty acid metabolism	3/24	57/8545	0.000515712	0.00670425	0.005157115
KEGG	hsa04920	Adipocytokine signaling pathway	3/24	70/8545	0.000941972	0.008685068	0.006680821

### 3.7 Gene Set Enrichment Analysis (GSEA)

**Fig 8:**
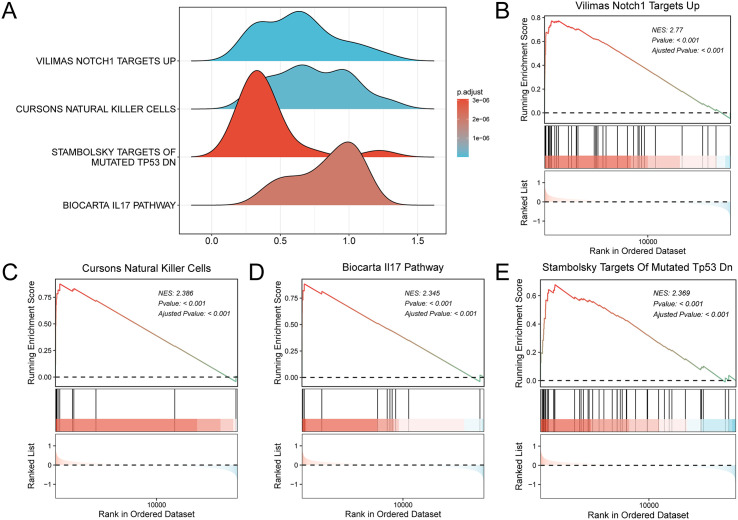
GSEA for the combined datasets. **(A)** Ridgeline plots of 4 biological functions of GSEA in the combined datasets; **(B-E)** Marked enrichment observed in the Vilimas notch1 targets up (B), Cursons natural killer cells (C), Biocarta IL17 pathway (D) and Stambolsky targets of mutated tp53 dn (E), respectively.

To elucidate the effects of the expression levels of all genes in the combined datasets for sepsis, GSEA was applied to study the associations between the expression levels of all genes and BP, CC, and MF. GSEA revealed the biological processes involved in all the genes (Fig 8A), the detailed results were shown in Table 3. The results indicated that all genes were significantly enriched in Vilimas notch1 targets (Fig 8B), Cursons natural killer cells (Fig 8C), the Biocarta IL17 pathway (Fig 8D), and Stambolsky targets of mutated tp53 dn (Fig 8E).

**Table 3:** Results of GSEA for the combined datasets.

ID	Set Size	Enrichment Score	NES	p value	p.adjust	q value
VILIMAS_NOTCH1_TARGETS_UP	51	0.774014732	2.769798696	1.00E-10	4.32E-09	3.29E-09
CURSONS_NATURAL_KILLER_CELLS	18	0.875312552	2.386180046	4.64E-09	1.51E-07	1.15E-07
STAMBOLSKY_TARGETS_OF_MUTATED_TP53_DN	47	0.678299937	2.36898639	1.29E-07	3.06E-06	2.33E-06
BIOCARTA_IL17_PATHWAY	15	0.883673272	2.344967753	8.02E-08	2.01E-06	1.53E-06

### 3.8 Construction of sepsis diagnostic model

**Fig 9:**
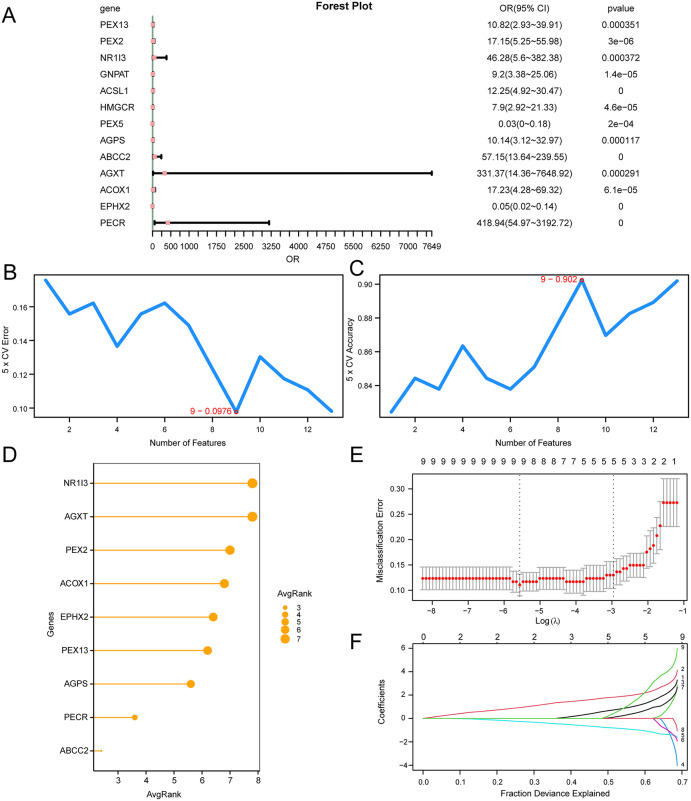
Diagnostic model of sepsis. **(A)** Forest map of the 13 hub genes; **(B)** The number of genes with the lowest error rate; **(C)** The number of genes with the highest accuracy; **(D)** The average importance ranking lollipop plot of the 9 genes; **(E)** Diagnostic model plot; **(F)** Variable trajectory plot.


Logistic regression analysis was conducted based on the 25 hub genes. The results were visually presented using a forest plot (Fig 9A). 13 hub genes were statistically significant in the logistic regression model, namely: *PEX13*, *PEX2*, *NR1I3*, *GNPAT*, *ACSL1*, *HMGCR*, *PEX5*, *AGPS*, *ABCC2*, *AGXT*, *ACOX1*, *EPHX2*, *PECR* (p value < 0.05). The SVM-RFE algorithm was applied to set up 5-fold cross validation based on the 13 hub genes. The average ranks of genes were calculated, and the genes number with the lowest error rate and highest accuracy rate of the model was obtained (Fig 9B and 9C). The results indicated that when the genes number was 9, the SVM model had the highest accuracy, and the top 9 genes in terms of average ranks were chosen for subsequent analysis, namely: *ABCC2*, *PECR*, *AGPS*, *PEX13*, *EPHX2*, *ACOX1*, *PEX2*, *NR1I3*, and *AGXT* (Fig 9D). A sepsis diagnostic model was established by LASSO regression analysis based on the 9 genes. The diagrams of LASSO regression model and LASSO variable trajectory were presented (Fig 9E and 9F). The results indicated that the 5 hub genes included in the LASSO regression model were key genes, namely *ABCC2*, *PECR*, *EPHX2*, *PEX2*, and *AGXT*. The LASSO risk score was calculated based on the risk coefficient, applying the following formula:

$$\begin{array}{c} \text{Risk}Score=ABCC\text{2}*(\text{0}.\text{932})+PECR*(\text{1}.\text{943})+EPHX\text{2}*(-\text{0}.\text{906})+PEX\text{2}*(\text{0}.\text{322})+AGXT*(\text{1}.\text{103}) \end{array}$$

### 3.9 Internal validation of sepsis diagnostic model

**Fig 10:**
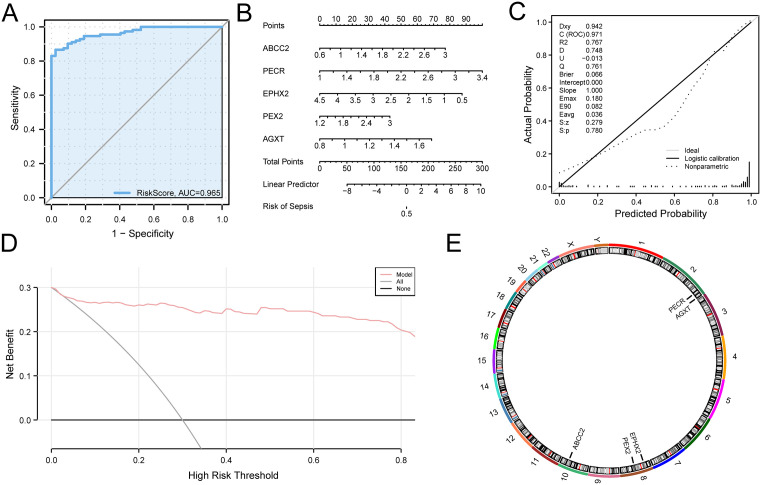
Diagnostic and validation of combined datasets. **(A)** ROC curves of risk score in the combined datasets; **(B)** Nomograms of key genes in the combined datasets; **(C-D)** Calibration curve plot (C) and DCA plot (D) of the sepsis diagnostic model based on risk score in the combined datasets; **(E)** Key genes of chromosome location map.

The ROC curve indicated that the risk score in the combined datasets showed a high accuracy between sepsis and normal groups (AUC > 0.9, Fig 10A). The nomogram indicated that PECR had a significantly higher utility and PEX2 had a significantly lower utility than other variables in the sepsis diagnostic model (Fig 10B). The calibration curve plot indicated that the calibration line represented by the dotted line deviated marginally slightly from the diagonal line of ideal model (Fig 10C). DCA was applied to assess the clinical effectiveness of sepsis diagnostic model, and the results suggested that the model line was more stable than that of all positive and negative models in a certain range, with a higher net benefit and better performance (Fig 10D). A chromosome localization map was used to present the locations of the 5 key genes on the human chromosome using the “RCircos” R package, and the results suggested that most of the key genes were located on chromosomes 2 and 8, namely: *PECR*, *AGXT*, *PEX2* and *EPHX2* (Fig 10E).

### 3.10 External validation of sepsis diagnostic model and Friends analysis

**Fig 11:**
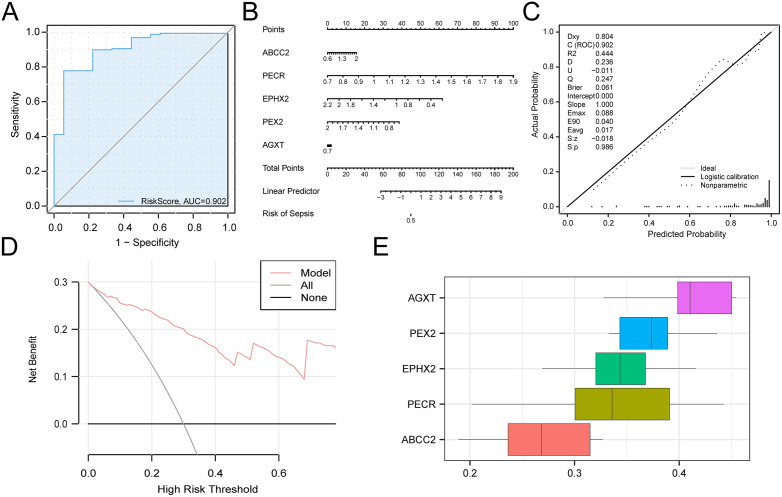
Diagnostic and validation of dataset GSE13904 and Friends analysis. **(A)** ROC curve of risk score in dataset GSE13904; **(B)** Nomogram of key genes in dataset GSE13904 in sepsis diagnostic model; **(C-D)** Calibration curve plot (C) and DCA plot (D) of the sepsis diagnostic model based on the risk score in dataset GSE13904; **(E)** Box plot of Friends analysis of key genes.

The ROC curve suggested that the expression levels of the risk score in dataset GSE13904 showed a high accuracy between sepsis and normal groups (AUC > 0.9, Fig 11A). The nomogram denoted that diagnostic utility was significantly higher on *PECR* and lower on *AGXT* compared with other variables. (Fig 11B). The calibration curve plot of sepsis diagnostic model indicated that the calibration line presented by the dotted line deviated from the diagonal line of ideal model (Fig 11C). The DCA indicated that the model line was stably higher than that of all positive and negative in a certain range, the model had a certain net benefit (Fig 11D). Friends analysis indicated that *AGXT* could play a significant role in sepsis (Fig 11E).

### 3.11 Correlation analysis of key genes

**Fig 12:**
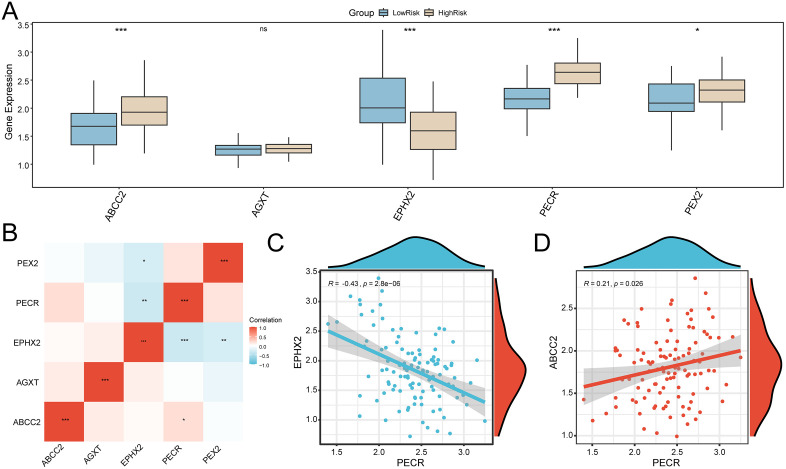
Correlation analysis of key genes. **(A)** Group comparison map of key genes in the high and low-risk group in the combined datasets; **(B)** Correlation heatmap of key genes; **(C)** Correlation scatter plot between *PECR* and *EPHX2*; **(D)** Correlation scatter plot between *PECR* and *ABCC2.* Red denotes a positive correlation, blue denotes a negative correlation, and the depth of color represents the strength of the correlation. Blue represents the low-risk group and yellow represents the high-risk group.

Comparison of the expression levels of the 5 key genes in high- and low-risk groups from the combined datasets indicated that *ABCC2*, *EPHX2*, and *PECR* gene*s* were significantly different (p < 0.001). *PEX2* expression levels differed significantly (p value < 0.05, Fig 12A). Correlation analysis was conducted, and a correlation heat map was visually presented (Fig. 12B). The results indicated that the strongest significant negative correlation exist between *PECR* and *EPHX2* (r value = −0.43, p value < 0.05, Fig 12C), and the strongest significant positive correlation exist between *PECR* and *ABCC2* (r value = 0.21, p value = 0.026, Fig 12D).

### 3.12 Gene Set Enrichment Analysis (GSEA)

**Fig 13:**
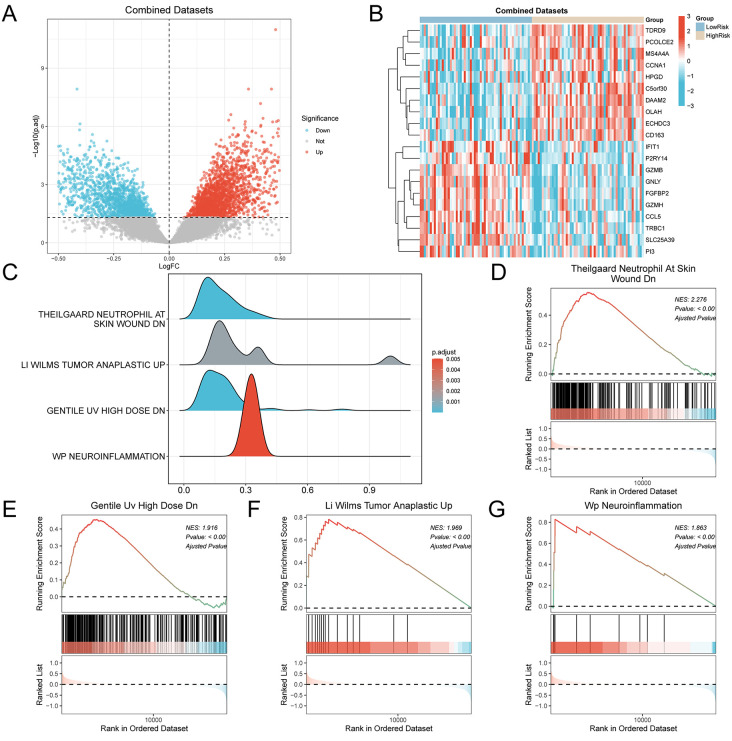
Differential gene expression analysis and GSEA for combined datasets. **(A-B)** Volcano map (A) and heat map (B) of DEGs analysis between the high- and low-risk groups of sepsis samples in the combined dataset; **(C)** Mountain map of the GSEA of the 4 biological functions of sepsis samples in the combined datasets; **(D-G)** Marked enrichment observed in the Theilgaard neutrophils at skin wound dn (D), Gentile uv high-dose dn (E), Li Wilm tumor anaplastic up (F), and neuroinflammation (G), respectively. In the heat map, blue represents the low-risk group and yellow represents the high-risk group, red represents high expression and blue represents low expression.

The examination of 3832 DEGs between the high- and low-risk groups uncovered 2124 genes exhibiting upregulation and 1708 genes demonstrating downregulation, the result was presented as a volcano plot (Fig 13A). The top 20 DEGs identified in |logFC| descending order were presented in a heat map (Fig 13B). GSEA revealed an association between the expression of all genes in the sepsis samples from the combined datasets and BP, CC, and MF (Fig 13C), the results were shown in Table 4, suggesting that all genes were significantly enriched in Theilgaard neutrophils at skin wound dn (Fig 13D), Gentile uv high dose dn (Fig 13E), Li Wilm tumor anaplastic up (Fig 13F), Neuroinflammation (Fig 13G).

**Table 4:** Results of GSEA for risk group.

ID	Set Size	Enrichment Score	NES	p value	p.adjust	q value
THEILGAARD_NEUTROPHIL_AT_SKIN_WOUND_DN	218	0.55649851	2.276397679	1.00E-10	8.40E-09	6.61E-09
LI_WILMS_TUMOR_ANAPLASTIC_UP	17	0.783011548	1.968970664	6.89E-05	0.001297845	0.001021057
GENTILE_UV_HIGH_DOSE_DN	273	0.457237953	1.916496527	4.75E-09	2.60E-07	2.04E-07
WP_NEUROINFLAMMATION	11	0.82829623	1.862657454	0.000333884	0.005036569	0.003962434

### 3.13 Construction of regulatory network

**Fig 14:**
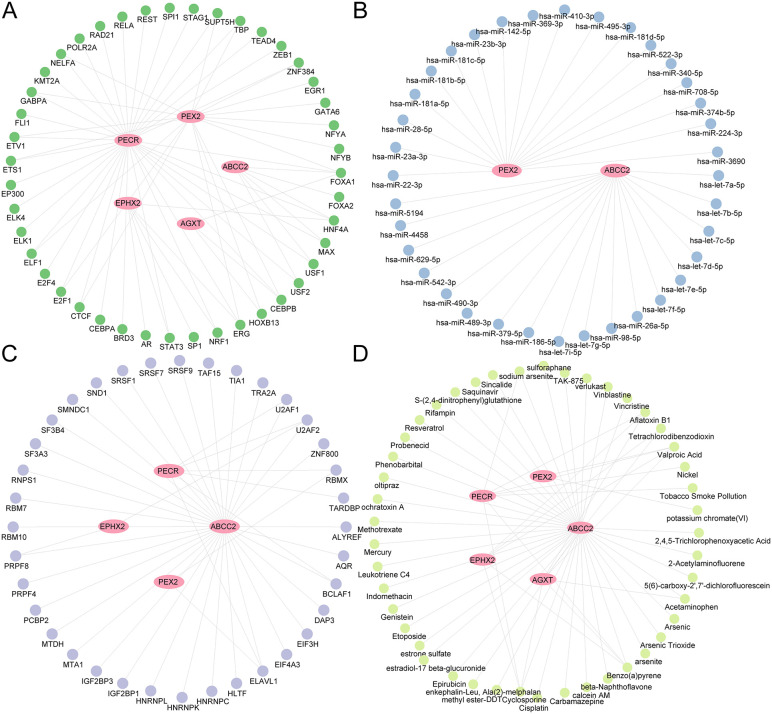
Regulatory network of key genes. (A) mRNA-TF regulatory network of key genes; **(B)** mRNA-miRNA regulatory network of key genes; **(C)** mRNA-RBP regulatory network of key genes; **(D)** mRNA-drug regulatory network of key genes. Pink is mRNA, dark green is TF, blue is miRNA, purple is RBP, and light green is Drug.

TFs combined with key genes were acquired, with 5 key genes and 43 TFs (Fig 14A). The miRNAs related to key genes were acquired, with 2 key genes and 36 miRNAs (Fig 14B). RBPs related to key genes were predicted, with 4 key genes and 36 RBPs (Fig 14C). The potential drugs or molecular compounds related to key genes were identified, with 5 key genes and 45 drugs or molecular compounds (Fig 14D). All regulatory networks were constructed and visualized using Cytoscape. All the detailed information were shown in S4–S7 Tables.

### 3.14 Prediction of protein domains

**Fig 15:**
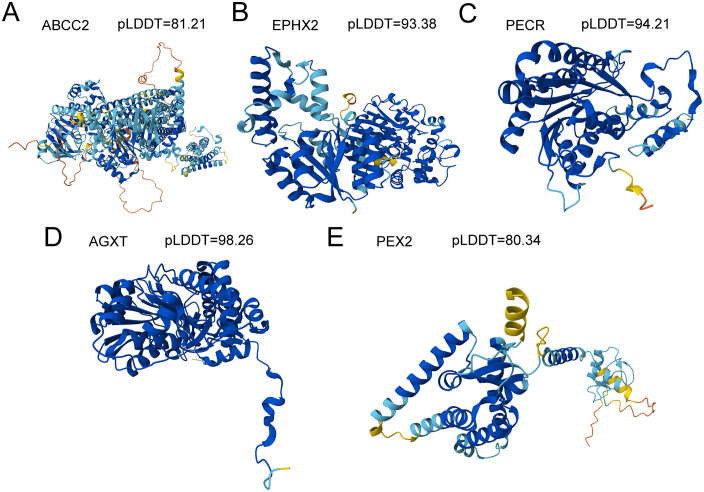
Protein structure of key genes. **(A-E)** Protein domains of key genes *ABCC2* (A), *EPHX2* (B), *PECR* (C), *AGXT* (D) and *PEX2* (E).

We acquired the protein structures of 5 key genes and displayed the results using AlphaFoldDB, namely *ABCC2* (Fig 15A), *EPHX2* (Fig 15B), *PECR* (Fig 15C), *AGXT* (Fig 15D), and *PEX2* (Fig 15E). The protein domains of the 5 key genes had high confidence (70 < pLDDT < 90).

### 3.15 Immune infiltration analysis of sepsis

**Fig 16:**
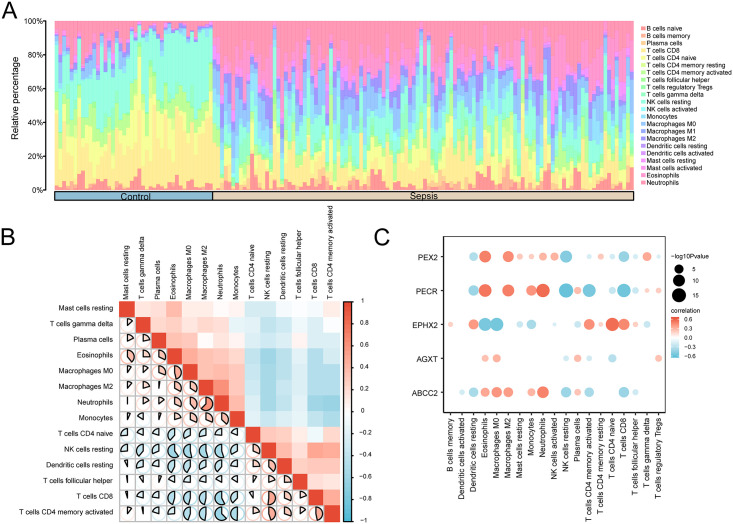
Immune infiltration analysis of the combined datasets. **(A)** Bar chart of the proportion of immune cells in the combined datasets; **(B)** Heatmap of correlations between immune cells; **(C)** Bubble plot of the correlation between the abundance of immune cell infiltration and key genes. Red represents positive correlation and blue represents negative correlation. The depth of the color represents the strength of the correlation. In the proportion bar chart, blue represents the control group and yellow represents the sepsis group.

The CIBERSORT algorithm was applied to compute the immune infiltration abundances of 22 immune cells in the combined datasets. A bar chart of the proportion of immune cells was plotted (Fig 16A). The correlation heat map suggested that most immune cells displayed a certain correlation, with the strongest positive correlation existed between Neutrophils and M2 macrophages (r = 0.626, p value < 0.05), with the strongest negative correlation existed between Neutrophils and CD4+ memory T-cell activation. (r value = −0.624, p value < 0.05) (Fig 16B). The results suggested *PECR* and M0 Macrophages had the strongest significant positive correlation (r = 0.667, p value < 0.05), *EPHX2* and M2 macrophages had the strongest significant negative correlation (r = −0.650, p value < 0.05, Fig 16C).

### 3.16 Validation of key genes

**Fig 17:**
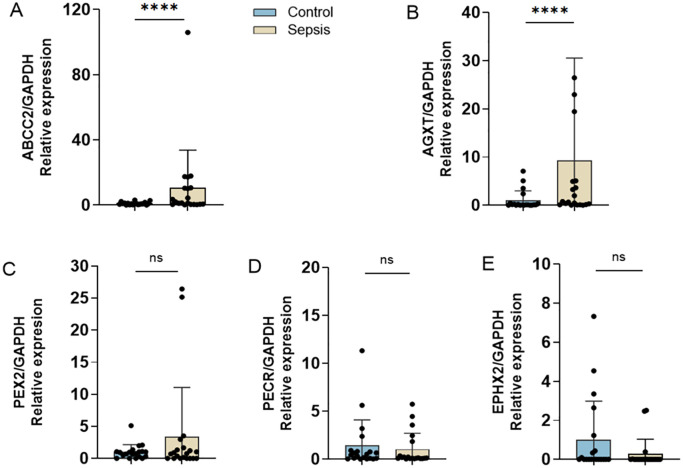
Validation of the key genes in sepsis patients. **(A-E)** The relative expression. levels of *ABCC2* (A), *AGXT* (B), *PEX2* (C), *PECR* (D) and *EPHX2* (E) between sepsis patients and controls by qRT-PCR. **** p value < 0.0001; ns, difference statistically not significant.

qRT-PCR experiments showed that the transcriptional expression levels of *ABCC2* and *AGXT* in the sepsis group were significantly higher than that in the control group (p value < 0.05, Fig 17A and 17B), thus further strengthening the reliability of the findings.

## 4. Discussion

Sepsis is a life-threatening clinical syndrome triggered by infection, characterized by severe metabolic disorders and multiple organ dysfunction. Sepsis has been established as a global health emergency by the World Health Organization (WHO) in 2017, with a nosocomial mortality rate of up to 20% [1]. To date, the lack of effective early diagnostic markers for sepsis poses a significant public health challenge. Elevated levels of bile acids in patients with sepsis are not only associated with liver dysfunction, but also can serve as potential biomarkers for predicting mortality risk [42]. Identifying genes related to bile acid metabolism in sepsis and developing a diagnostic model are of great significance for the early sepsis screening and finding potential molecular targets. This study aims to improve our understanding of bile acid metabolism in the pathogenesis of sepsis through combining systematic analysis of large-scale datasets.

This study yielded multiple critical outcomes, including the identification of 25 bile acid metabolism-related hub genes. Among them, the expression levels of 14 hub genes in both sepsis and control groups were statistically significant, certain genes had high accuracy in differentiating sepsis from controls, with *PECR* and *EPHX2* having high AUC values (AUC > 0.9). LASSO regression and SVM-RFE technologies were conducted to develop a diagnostic model, identifying 5 key genes: *ABCC2*, *PECR*, *EPHX2*, *PEX2*, and *AGXT*. The diagnostic model was validated in both the combined datasets and dataset GSE13904, both of which showed good accuracy and resolution. Based on this, standardized nomograms were applied to assess the weight and contribution of each key gene to the diagnostic model. The decision curve analysis indicated that using key genes for risk prediction could provide substantial clinical benefits. In addition, the calibration curve further corroborated the considerable predictive accuracy of the bias-corrected model. This model provided a new tool for the early screening for sepsis and holds promise for improving sepsis outcomes. qRT-PCR revealed significant increase on *ABCC2* and *AGXT* in sepsis versus controls, the reliability and biological relevance of the key genes were further verified at the experimental level and the results were generally consistent with the bioinformatics analysis. We believe that the 5 key genes hold crucial value as diagnostic and treatment biomarkers for sepsis, and future studies should explore the functions of key genes to further clarify the pathogenesis of sepsis.

*ABCC2* encodes multidrug resistance related protein 2 (MRP2). As an excretion transport protein, MRP2 mediates the excretion of multiple drugs and metabolites, and is especially involved in transporting bilirubin to the bile tubules. MRP2 promotes the excretion of bile salt conjugates from kidneys during cholestasis, thereby maintaining bile acid homeostasis [43]. *PECR* encodes peroxisomal trans-2-enoyl-CoA reductase (PECR). PECR is a peroxisomal protein that is involved in fatty acid synthesis. This protein plays a critical role in fat synthesis and late triglyceride development. Bta-miR-124a participates in lipid metabolism by directly downregulating the expression levels of *PECR* and impacting downstream *ELOVL2* expression, thus regulating the production of triglycerides and free fatty acids (FFA) [44]. *EPHX2* encodes soluble epoxide hydrolase(sEH). sEH acts as both phosphatase and epoxide hydrolase activities, and participates in the lipid metabolism through decomposing epoxy fatty acids into 1, 2-diols [45]. *PEX2* is a core regulatory gene in the peroxisome biogenesis pathway that functions to encode peroxisomal membrane proteins. *PEX2* acts as an intracellular fatty acid sensor to maintain fatty acid homeostasis through reactive oxygen species-mediated feedback regulation. Specifically, *PEX2* regulates triglyceride lipase (ATGL) protein levels through specific polyubiquitination, peroxisome-derived reactive oxygen species β-oxidation stably regulate PEX2 protein levels through disulfide bonds, thereby promoting ATGL degradation [46]. *AGXT* encodes alanine-glyoxylate aminotransferase (AGT). AGT is a liver-specific enzyme that primarily functions to convert glyoxylate into glycine. Oxalic acid metabolic disorders frequently occur in patients with metabolic dysfunction-associated steatohepatitis, with downregulated levels of *AGXT*. Overexpression of *AGXT* in the liver could reduce the levels of oxalic acid and attenuate hepatic steatosis by inducing fatty acid oxidation [47].

GO and KEGG enrichment analyses revealed significant regulation of diverse biological metabolic pathways related to sepsis, including fatty acid metabolic processes, bile acid metabolic processes, and peroxisome pathways. Up-regulated levels of fatty acid synthetase occur in patients with sepsis. Excessive fatty acids can cause mitochondrial dysfunction, subsequent activation of the cGAS-STING pathway trigger cell pyroptosis, and further aggravate sepsis-induced lung injury [48]. Bile acids regulate metabolism and immunity by activating farnesoid X receptor (FXR) and G protein-coupled bile acid receptor 1 (GPBAR1) [49]. High levels of bile acids exist in patients with septic shock complicated with liver failure and bile acids participate in the induction of immunosuppression by activating GPBAR1 [42]. Overactivation of NLRP3 inflammasomes is considered a vital contributor to the pathogenesis of septic damage, and bile acids, in combination with ATP activate the inflammasome NLRP3. FXRs have been identified as negative regulators of the NLRP3 inflammasome through direct interactions with NLRP3 to alleviate pathological damage caused by sepsis [6]. Peroxisome proliferator-activated receptors (PPARs) are essentially nuclear hormone receptors that are activated by fatty acids and their derivatives. Activated PPARɑ could promote peroxisomal fatty acid β-oxidation through initiating the expression of key enzymes for fatty acid oxidation (CPT1 and ACOX1), resulting in reduced fat accumulation in the liver. PPARɑ regulates lipoprotein metabolism through modulating apolipoprotein expression, thereby increasing plasma high-density lipoprotein cholesterol levels and lessening low-density lipoprotein cholesterol levels [50]. These findings demonstrate that BAMRDEG involved in sepsis progression exert synergistic effects through multiple pathways.

Immune cell infiltration analysis revealed complex interactions between different types of immune cells during sepsis. Our findings are in agreement with previous studies, suggesting that significantly elevated levels of infiltration of neutrophils and macrophages occur during sepsis [51]. In sepsis, the activation of innate immune cells boosts the host defense against pathogens by inducing systemic inflammatory responses. Neutrophil levels are positively correlated with M2 macrophages, but negatively correlated with CD4 memory T cell activation. Sepsis progression is also correlated with significant lymphocytopenia accompanied by reduced counts of CD8+ and CD4+ T cells. Specific neutrophil subsets suppress lymphocyte function, leading to immunosuppression [52]. Mounting evidence supports immunosuppression as a major cause of sepsis-related death, particularly T-cell exhaustion [53]. Furthermore, the expression levels of key genes were significantly associated with the abundance of different types of immune cells. For example, *PECR* expression presented the strongest positive correlation with the abundance of macrophages M0 cells, whereas *EPHX2* expression presented the strongest negative correlation with the abundance of M2 macrophages. These findings further support the role of these genes as sepsis biomarkers and provide new insights into potential immune targets. Thus, we speculate that these key genes contribute to the occurrence and progression of sepsis via modulating multiple immune cells. It should be noted that the immune cell composition analysis in this study is based on the deconvolution results of peripheral blood transcriptomes, reflecting the relative trends of changes in different immune cell subsets. Due to the complex cellular composition of whole blood samples, there may be overlapping transcriptional signals between some phenotypically similar cell populations, and the estimation of low-abundance cell subsets may also be affected to some extent. Therefore, the related results are more suitable as auxiliary immunological clues to support the potential link between key genes and immune imbalance, and their specific cytological significance still requires further validation through flow cytometry or other independent methods.

This research has multiple advantages in terms of research design and methodology. Firstly, this study integrated three independent GEO transcriptome datasets (GSE28750, GSE95233, and GSE13904). The batch effects were effectively abrogated by the sva algorithm, making the data range comparable across the different platforms. This integrated analysis strategy significantly enhances the stability of the model and the universality of the results while expanding the sample size. Secondly, the research adopted a multi-level algorithm screening process, including the cytoHubba five-algorithm identification of hub genes, Logistic regression, SVM-RFE feature selection and LASSO regression model construction, gradually optimizing and screening out the most representative key genes (*ABCC2*, *PECR*, *EPHX2*, *PEX2* and *AGXT*) minimizes the common overfitting risk in bioinformatics analysis. Furthermore, the diagnostic model has excellent generalization ability, with high diagnostic accuracy both in the combined dataset and dataset GSE13904（AUC > 0.9). Combined with immune infiltration analysis and multi-level regulatory network construction, this study systematically revealed the potential role of key genes in the pathogenesis of sepsis from the perspectives of transcriptome immunology and molecular regulation, providing multi-angle support for the biological rationality of the results.

Although the results of this study are reliable and innovative, there are still certain limitations. Firstly, the study mainly relies on public database data and the sample sources are heterogeneous. The datasets do not cover different pathogen types, disease stages, and age groups, which may affect the comprehensiveness of the results. Secondly, this study lacks the validation of cell experiments and animal models, and the expression and function of key genes should be further verified by Western blot and immunohistochemistry in the future. In addition, due to the lack of long-term follow-up information in the relevant datasets, it is still impossible to evaluate the applicability of the model in disease prognosis prediction. Finally, although immune deconvolution analysis provides clues for understanding the relationship between key genes and immune abnormalities in sepsis, the conclusions of this section still need to be further verified in combination with immune quantification methods or experimental data that are more applicable to blood samples. Therefore, the interpretation of immune cell changes in this study primarily remains at the level of transcriptomic inference. Its significance lies in providing direction for subsequent mechanistic research, rather than serving as the final basis for determining immune phenotypes. Overall, the clinical translation of the model still needs to be further verified in a multicenter, large-sample prospective study to ensure its feasibility and reliability in actual clinical diagnosis.

## 5. Conclusion

In summary, bioinformatics methods revealed *ABCC2*, *PECR*, *EPHX2*, *PEX2* and *AGXT* as key BAMRDEGs that could support the diagnosis of sepsis and innovatively constructed an efficient diagnostic model. These findings not only deepen our understanding of the mechanisms involved in sepsis but also lay the foundation for the development of more precise and targeted interventions.

## Supporting information

S1 File Supplementary Table 1. (CSV)

S2 File Supplementary Table 2. (CSV)

S3 File Supplementary Table 3. (TSV)

S4 File Supplementary Table 4. (CSV)

S5 File Supplementary Table 5. (CSV)

S6 File Supplementary Table 6. (CSV)

S7 File Supplementary Table 7. (TXT)
