# Supplementary material for: Bioinformatics analysis reveals the association of bile acid metabolism-related genes with sepsis
Source: PLoS One. 2026 Jul 10;21(7):e0352431. doi: 10.1371/journal.pone.0352431 (PMC13354078; doi:10.1371/journal.pone.0352431)
Supplement: Attachment — Submitted filename: Response to Reviewers.doc [file pone.0352431.s009.doc]

Dear Editors and Reviewers:

We thank the reviewers for reviewing the manuscript and for their constructive comments during the review process. Your comments, along with those of the other reviewers, are extremely important to our article. We greatly appreciate your insights, which have significantly contributed to enhancing the quality of our work. In this revision, we have prepared a detailed point-by-point response letter for you and the other valued reviewers. This letter acknowledges your feedback and clearly outlines the revisions we have made. In response to the reviewers' comments, we have made extensive revisions to the manuscript. If there are any further modifications you would like us to consider, we would be more than willing to accommodate them, and we genuinely appreciate your assistance. [PLOS One] is a highly esteemed journal, and we hope that our manuscript can be considered for publication. Thank you very much for your support. We would like to express our sincere gratitude to you and the reviewers for your valuable comments on our manuscript. In light of the thorough and insightful feedback provided, we have revised the manuscript accordingly. We have added relevant content to address the reviewers' suggestions, and we have rewritten several sections to ensure clarity and completeness. We hope that these modifications meet the reviewers' expectations and enhance the overall quality of the manuscript.

The authors need to address the following points before being considered for publication. Responds to the reviewer’s comments:

1.When identifying the differentially expressed genes (DEGs), why used p-values without any adjustment for multiple testing and a threshold of 0 for |logFC|?

Reply to the reviewers: Thank you for carefully reviewing our manuscript and providing valuable comments. When we initially screened differentially expressed genes (DEGs), the threshold was set as |logFC| > 0 and p < 0.05, mainly for the following considerations: Firstly, the aim of this study is to comprehensively explore as many candidate genes related to bile acid metabolism associated with sepsis as possible, in order to provide a sufficient gene set foundation for subsequent protein interaction networks (PPIs), enrichment analysis, and machine learning model construction. If overly strict thresholds (such as high |logFC| or multiple corrected p values) are adopted in the initial screening stage, it may lead to the omission of some genes with potential biological significance but relatively small expression variations, thereby affecting the integrity of the overall network analysis. In the subsequent analysis, we gradually narrowed down the candidate scope through multi-level validation (including CytoHubba algorithm screening, LASSO regression, SVM-RFE cross-validation) to ensure the robustness and reliability of the final key genes from statistical and biological perspectives. In addition, similar initial screening criteria have been used in genome-wide exploratory study to ensure broad coverage of the analysis (PMID: 39021997). In this article, although a threshold of |logFC| > 1 was used in the preliminary analysis, the threshold was adjusted to |logFC| > 0 and p < 0.05 when identifying ARDEGs, indicating that the threshold selection for fold-change in differential analysis can be appropriately broadened so as to incorporate as many DEGs as possible. We fully agree with the reviewers' suggestions, in future further research, multiple hypothesis testing corrections (such as FDR) and a more reasonable threshold for logFC will be considered in the initial screening phase to enhance the rigor of the screening criteria and the persuasiveness of the results. Thank you again for your valuable suggestions.

2.For the training set, why combined the two datasets with small sample sizes. Plus, the authors barely mentioned to how to pre-process the gene expression data (if they directly downloaded the expression matrix from the GEO database, the used pre-process procedures may differ, which would significantly affect the results. Also, please give more details on how to deal with batch effect when combining the datasets.

Reply to the reviewers: Thank you for carefully reviewing our manuscript and providing valuable comments. In this study, both the GSE28750 and GSE95233 are derived from the same platform (GPL570) and have the same organizational source.To ensure the statistical performance and the robustness of the model, the two are combined as a training set. In terms of data preprocessing, firstly, the probe numbers were converted into gene symbols according to the annotation file of the corresponding platform of each dataset.For datasets from different platforms, after probe conversion only the intersecting genes were retained to ensure data consistency. Subsequently, the normalizeBetweenArrays function in the R package limma was used for standardization processing to eliminate systematic errors between samples. Finally, the batch effect correction was done using the ComBat function in the R-package sva, and the correction effect was verified by principal component analysis (PCA), and the results showed that the batch difference was effectively eliminated. We have further supplemented the unified preprocessing process description for the original data in the revised draft, so that readers can have a clearer understanding of the data integration and correction process.For specific details of the modifications, please refer to page 4-5 and line 85-100.

Thank you again for your valuable suggestions.

3. No experimental validation has been conducted, please perform such a validation.

Reply to the reviewers: Thank you for carefully reviewing our manuscript and providing valuable comments. We fully understand your suggestions regarding experimental verification. We have added qRT-PCR experiments in the revised draft to validate the expression changes of key genes in sepsis samples. Specifically, we examined the relative expression levels of five key genes (*ABCC2*，*AGXT*，*PEX2*，*PECR*，*EPHX2*) in the sepsis group and the control group, and the results showed that the transcriptional expression levels of *ABCC2* and *AGXT* in the sepsis group were significantly higher than that in the control group, and there were no statistically significant differences between the two groups in *PEX2*, *PECR* and *EPHX2*. The reliability and biological relevance of the key genes were further verified at the experimental level and results are generally consistent with the bioinformatics analysis results. We have supplemented the qRT-PCR experimental data in the results section of the revised draft, and expounded on its consistency with the bioinformatics analysis results and its biological significance in the discussion section. Thank you again for your valuable suggestions.

4.The bioinformatics methods used are very conventional, please justify in each step the conventional methods instead of more novel methods were used.

Reply to the reviewers: Thank you for carefully reviewing our manuscript and providing valuable comments. The major strength of bioinformatics methods adopted in this study pertains to its robustness, interpretability and reproducibility. In response to the questions you raised, we have explained the scientific basis and suitability of selecting traditional algorithms for each step in the revised draft. Specifically, the data in this study is derived from multi-platform chip data. Classical algorithm “sva” and “limma” have mature theoretical foundations and widely verified reliability in such data processing. The STRING database and the cytoHubba plugin were applied to PPI network analysis to ensure the stability and comparability of the results. GO/KEGG and GSEA are internationally recognized functional annotation systems, suitable for systematic analysis of disease mechanisms. Logistic Regression, SVM-RFE and LASSO models have good generalization and biological explanatory power in the analysis of small sample high-dimensional data. The CIBERSORT algorithm has been widely verified in immune infiltration analysis in blood samples and can accurately reflect the composition of immune cells. Given the biological significance and clinical translational potential of the results focused on in this study, we believe that adopting conventional bioinformatics methods can better ensure the stability and repeatability of the analysis. In subsequent research, we will further explore the application potential of emerging algorithms such as deep learning to expand the depth and breadth of the analysis.

For specific details of the modifications, please refer to methodology section.

Thank you again for your valuable suggestions.

5. Please a more detailed discussion on the strengths and limitations of current study, particularly the clinical significance of constructing such a gene signature.

Reply to the reviewers: Thank you for carefully reviewing our manuscript and providing valuable comments. We have provided a more systematic elaboration on the advantages and limitations of the research as well as the clinical significance of the constructed gene characteristics. In the revised draft, we have further emphasized multiple advantages of this study, including multi-dataset integration，batch effect control and multi-algorithm joint screening for key genes (SVM-RFE algorithm and LASSO regression), as well as the evaluation of model performance through internal and external validation. Meanwhile, we have supplemented the objective limitations of this study in terms of data source，sample size，lack of experimental validation and clinical translation, and further clarified the application prospects of this model in early diagnosis of sepsis, individualized risk assessment and discovery of potential therapeutic targets. For specific details of the modifications, please refer to page 47-48 and line 654-681.Thank you again for your valuable suggestions.

Journal Requirements:

1. When submitting your revision, we need you to address these additional requirements.

Please ensure that your manuscript meets PLOS ONE's style requirements, including those for file naming. The PLOS ONE style templates can be found at

https://journals.plos.org/plosone/s/file?id=wjVg/PLOSOne_formatting_sample_main_body.pdf and

<https://journals.plos.org/plosone/s/file?id=ba62/PLOSOne_formatting_sample_title_authors_affiliations.pdf>

Reply to the editors: Thank you for carefully reviewing our manuscript and providing valuable comments. In strict accordance with the requirements of the PLOS ONE official template, we have comprehensively adjusted the title page, abstract, body level, chart naming and file naming format in the new revised draft, making the manuscript fully complied with the journal specifications in terms of structure and layout. Thank you again for your valuable suggestions.

2. Thank you for stating the following financial disclosure:

This study is sponsored by the National Natural Science Foundation of China (General Program No.82171738) and the Special Funds for Health Science and Technology Development of Nanjing City (No. YKK20145). Please state what role the funders took in the study. If the funders had no role, please state: "The funders had no role in study design, data collection and analysis, decision to publish, or preparation of the manuscript." If this statement is not correct you must amend it as needed. Please include this amended Role of Funder statement in your cover letter; we will change the online submission form on your behalf.

Reply to the editor: Thank you for carefully reviewing our manuscript and providing valuable comments. The author(s) received no specific funding for this work, the previously incorrect expressions have been corrected. We have updated the relevant instructions in the manuscript. Thank you again for your valuable suggestions.

3.We are unable to open your Supporting Information file“Table S3-string_interactions_short.tsv”. Please kindly revise as necessary and re-upload.

Reply to the editors: Thank you for carefully reviewing our manuscript and providing valuable comments. Regarding the issue you mentioned that the “Supporting Information” file cannot be opened, we have re-exported and uploaded the file in the new revised draft, uniformly using the XLS format to ensure that it can be opened normally in common systems and software environments. Thank you again for your valuable suggestions.

4. If the reviewer comments include a recommendation to cite specific previously published works, please review and evaluate these publications to determine whether they are relevant and should be cited. There is no requirement to cite these works unless the editor has indicated otherwise.

Reply to the editors: Thank you very much for reminding us. Such scenario was not encountered in the current study. Thank you again for your valuable suggestions.

Reviewers' comments:

Reviewer's Responses to Questions

Comments to the Author

1. Is the manuscript technically sound, and do the data support the conclusions?

The manuscript must describe a technically sound piece of scientific research with data that supports the conclusions. Experiments must have been conducted rigorously, with appropriate controls, replication, and sample sizes. The conclusions must be drawn appropriately based on the data presented.

Reply to the reviewers: Thank you for carefully reviewing our manuscript and providing valuable comments. We have conducted a comprehensive assessment of the recommended literature based on your suggestions and selected the best ones for citation in combination with the relevance of the research topic, methods and content, in order to further improve the theoretical and methodological support of the manuscript. For literature with significant differences in research directions or methods, we do not include them in the scope of citation to maintain the concentration and logical consistency of the discussion. Thank you again for your valuable suggestions.

Reviewer #1: Yes

2. Has the statistical analysis been performed appropriately and rigorously?

Reviewer #1: Yes

3. Have the authors made all data underlying the findings in their manuscript fully available?

The PLOS Data policy requires authors to make all data underlying the findings described in their manuscript fully available without restriction, with rare exception (please refer to the Data Availability Statement in the manuscript PDF file). The data should be provided as part of the manuscript or its supporting information, or deposited to a public repository. For example, in addition to summary statistics, the data points behind means, medians and variance measures should be available. If there are restrictions on publicly sharing data—e.g. participant privacy or use of data from a third party—those must be specified.

Reviewer #1: Yes

4. Is the manuscript presented in an intelligible fashion and written in standard English?

PLOS ONE does not copyedit accepted manuscripts, so the language in submitted articles must be clear, correct, and unambiguous. Any typographical or grammatical errors should be corrected at revision, so please note any specific errors here.

Reviewer #1: No

5. Review Comments to the Author

Please use the space provided to explain your answers to the questions above. You may also include additional comments for the author, including concerns about dual publication, research ethics, or publication ethics. (Please upload your review as an attachment if it exceeds 20,000 characters)

Reviewer #1: 1.The article contains some grammatical errors and is overly verbose; it is recommended to simplify and polish the text.

Reply to the reviewers: Thank you for carefully reviewing our manuscript and providing valuable comments. We have entrusted a professional proofreading agency to conduct a comprehensive language editing for the manuscript to ensure that the language expression is accurate and luent, and meets academic standards. Thank you again for your valuable suggestions.

2. Is the threshold |logFC| > 0 in section 2.2 overly lenient? Please briefly explain the criteria and rationale for this setting.

Reply to the reviewers: Thank you for carefully reviewing our manuscript and providing valuable comments. When we initially screened differentially expressed genes (DEGs), we adopted a threshold of logFC 0 and p 0.05, mainly based on the consideration of exploratory research. This study aims to comprehensively explore candidate genes related to bile acid metabolism in sepsis. Therefore, in the initial screening stage, we hope to retain potentially biologically significant genes with small expression changes as many as possible, in order to avoid missing key regulatory factors and provide a more complete gene set for the subsequent PPI network construction, enrichment analysis, machine learning model establishment, and immune infiltration analysis.

Since we subsequently combined multi-layer screening and cross-validation strategies (including cytoHubba algorithm, SVM-RFE algorithm, and LASSO regression), this initially loose threshold does not reduce the reliability of the results. Instead, it helps to gradually screen out the most stable and biologically significant core genes in the multi-stage analysis.

In addition, a high-quality study has also adopted the same initial screening criteria under similar data conditions to ensure broad coverage and systematization of the analysis (PMID: 39021997). We fully agree with the reviewers' suggestions and will consider introducing stricter logFC thresholds or multiplex test calibration criteria in the initial screening stage in subsequent studies to further enhance the rationality of the screening criteria and the persuasiveness of the results. Thank you again for your valuable suggestions.

3. It is recommended to incorporate additional clinical validation experiments to enhance the accuracy and reliability of the analytical results.

Reply to the reviewers: Thank you for carefully reviewing our manuscript and providing valuable comments. In the revised draft, we have supplemented the qRT- PCR experiment to detect the relative expression levels of five key genes (*ABCC2*, *AGXT*, *PEX2*, *PECR*, and *EPHX2*) in peripheral blood samples from patients with sepsis and healthy controls. qRT-PCR experiments showed that the transcriptional

expression levels of *ABCC2* and *AGXT* in the sepsis group were significantly higher

than that in the control group (p value < 0.05). There were no statistically significant

differences between the two groups in *PEX2*, *PECR* and *EPHX2* (p value > 0.05). The overall trend is consistent with the results of our bioinformatics analysis, and the reliability of the key genes and the credibility of the analysis conclusions have been preliminarily verified at the experimental level.

We are also fully aware that the current validation is still in the initial exploration stage. In the future, we plan to conduct more systematic validation studies on the basis of expanding the sample size and combining multi-center clinical samples, including protein level detection and functional experiments, in order to further enhance the clinical accuracy and wide applicability of the research results. Thank you again for your valuable suggestions.

6. PLOS authors have the option to publish the peer review history of their article (what does this mean?). If published, this will include your full peer review and any attached files.

If you choose “no”, your identity will remain anonymous but your review may still be made public.

Do you want your identity to be public for this peer review? For information about this choice, including consent withdrawal, please see our Privacy Policy.

Reviewer #1: No

[NOTE: If reviewer comments were submitted as an attachment file, they will be attached to this email and accessible via the submission site. Please log into your account, locate the manuscript record, and check for the action link "View Attachments". If this link does not appear, there are no attachment files.]

To ensure your figures meet our technical requirements, please review our figure guidelines: https://journals.plos.org/plosone/s/figures

You may also use PLOS’s free figure tool, NAAS, to help you prepare publication quality figures: https://journals.plos.org/plosone/s/figures#loc-tools-for-figure-preparation.

NAAS will assess whether your figures meet our technical requirements by comparing each figure against our figure specifications.

Reply to the reviewers: Thank you for carefully reviewing our manuscript and providing valuable comments. We have strictly followed the image technical specifications of PLOS journals and the requirements of the NAAS tool to systematically check and adjust the format, resolution, color mode and font identification of all images to ensure compliance with publication standards and improve the quality of image display. Thank you again for your valuable suggestions.

In compliance with data protection regulations, you may request that we remove your personal registration details at any time. (Remove my information/details). Please contact the publication office if you have any questions.

We are grateful for the invaluable feedback provided by the reviewers, and we look forward to the opportunity to improve our manuscript in response to their guidance.
