# Supplementary material for: Bioinformatics analysis reveals the association of bile acid metabolism-related genes with sepsis
Source: PLoS One. 2026 Jul 10;21(7):e0352431. doi: 10.1371/journal.pone.0352431 (PMC13354078; doi:10.1371/journal.pone.0352431)
Supplement: Attachment — Submitted filename: Response to Reviewers 5.27.doc [file pone.0352431.s010.doc]

Dear Editors and Reviewers:

We thank the reviewers for reviewing the manuscript and for their constructive comments during the review process. Your comments, along with those of the other reviewers, are extremely important to our article. We greatly appreciate your insights, which have significantly contributed to enhancing the quality of our work. In this revision, we have prepared a detailed point-by-point response letter for you and the other valued reviewers. This letter acknowledges your feedback and clearly outlines the revisions we have made. In response to the reviewers' comments, we have made extensive revisions to the manuscript. If there are any further modifications you would like us to consider, we would be more than willing to accommodate them, and we genuinely appreciate your assistance. [PLOS One] is a highly esteemed journal, and we hope that our manuscript can be considered for publication. Thank you very much for your support. We would like to express our sincere gratitude to you and the reviewers for your valuable comments on our manuscript. In light of the thorough and insightful feedback provided, we have revised the manuscript accordingly. We have added relevant content to address the reviewers' suggestions, and we have rewritten several sections to ensure clarity and completeness. We hope that these modifications meet the reviewers' expectations and enhance the overall quality of the manuscript.

The authors need to address the following points before being considered for publication. Responds to the reviewer’s comments:

Reviewer #2: This study attempts to identify bile acid metabolism-related diagnostic markers for sepsis using a standard bioinformatics pipeline followed by qRT-PCR validation. While the topic has some interest, I have three major concerns regarding methodology, analysis appropriateness, and validation completeness. A major revision is required before the manuscript can be considered for publication.

1. The authors used |logFC| > 0 and raw p < 0.05 without multiple testing correction to identify DEGs, claiming an “exploratory” approach to avoid missing biologically relevant genes with small effect sizes. This rationale is insufficient. No evidence is provided that genes included only under this loose threshold actually contribute to the final diagnostic model or improve its performance. To justify this choice, the authors must perform a sensitivity analysis comparing the current loose threshold with a more rigorous one (e.g., |logFC| > 0.5, FDR < 0.05). The comparison should include at least: (a) the number of DEGs and BAMRDEGs identified; (b) the hub genes selected; (c) the key genes selected by LASSO+SVM-RFE; and (d) the diagnostic AUC in both training and validation datasets. If the results are similar, the loose threshold may be acceptable; if not, the stricter criteria must be adopted. This analysis should be presented as a supplementary table or figure.

Reply to the reviewers: Thank you for carefully reviewing our manuscript and providing valuable comments. We fully understand your concerns regarding the threshold settings for the initial screening of DEGs. Regarding the use of |logFC| > 0 and raw P < 0.05 as the preliminary criteria for DEGs in this study, we would like to further clarify that this step was primarily employed for exploratory screening of candidate genes, rather than serving as the sole basis for final key genes identification or diagnostic model construction. Since the peripheral blood transcriptome data included in this study were derived from different independent cohorts, and sepsis exhibits strong clinical and biological heterogeneity, some biological changes related to bile acid metabolism are more likely to manifest as expression changes with small effect sizes but stable directionality. In this research context, if a high fold change threshold is applied at the initial screening stage, it may prematurely exclude some candidate genes with potential biological significance.

More importantly, the final results of this study are not directly based on the DEGs screened under all lenient thresholds. Instead, after the initial screening, further multi-round contraction analyses were conducted, including intersection with bile acid metabolism-related gene sets, PPI network screening, intersection analysis of five algorithms from cytoHubba, Logistic regression, SVM-RFE, and LASSO regression. In other words, the primary purpose of the initial DEG screening is to enhance the sensitivity in the candidate signal discovery phase, while the final key genes and diagnostic model are established based on continuous screening, cross-optimization and independent validation. Therefore, we believe that the current threshold is more suitable as a pre-screening criterion for the candidate gene discovery phase in this study, rather than the sole criterion for directly determining the final conclusions.

Furthermore, this study did not stop at the level of simple differential analysis, instead, further validated the diagnostic performance of the model in the dataset GSE13904, and experimentally verified the expression trends of key genes using qRT-PCR. This analytical framework of "exploratory initial screening + multi-round feature constriction + external validation + experimental validation" reduces the impact of threshold settings in individual steps on the stability of the final results to a certain extent. Similarly, some bioinformatics studies have also adopted a screening strategy of |logFC| > 0 and P < 0.05 during the candidate genes exploration stage, followed by further screening of key molecules through network analysis or machine learning methods (PMID: 34955918, PMID: 39916852, PMID: 40750912). Thank you again for your valuable suggestions.

2. The authors used CIBERSORT to estimate immune cell infiltration. CIBERSORT was originally developed and validated for tumor tissue transcriptomes, where cell type composition is markedly different from peripheral blood. Its application to whole blood samples is not standard and may produce unreliable estimates, especially for rare or closely related cell populations. The authors should either: (a) provide validation or cite studies that have successfully applied CIBERSORT to blood samples with similar immune cell deconvolution goals; or (b) use an alternative method specifically designed for blood, such as ImmuCellAI, xCell, or a reference-based deconvolution approach with a blood-derived signature matrix. Alternatively, the authors could remove the immune infiltration analysis if it does not directly support the main conclusion.

Reply to the reviewers: Thank you for carefully reviewing our manuscript and providing valuable comments. We greatly appreciate your attention to the applicability of immune infiltration analysis methods. Regarding the use of CIBERSORT for estimating immune cell composition in the peripheral blood transcriptome in this study, we have carefully re-examined the relevant methodological basis. We understand your concern that this method may have limitations in the whole blood samples, which is crucial for the rigor of result interpretation.

Meanwhile, we would like to provide an appropriate explanation regarding this issue. CIBERSORT is not an immune infiltration tool developed exclusively for tumor tissues. Its original method is based on the LM22 leukocyte signature matrix, primarily used to estimate the relative abundance of 22 types of human immune cell subsets in complex mixed samples, the establishment of methodology itself includes the modeling and validation of the expression characteristics related to leukocytes(PMID: 25822800). Therefore, from a methodological perspective, simply interpreting it as a tool "only applicable to tumor tissues" is not entirely accurate. Based on this, we believe that using CIBERSORT for preliminary immune deconvolution analysis in peripheral blood transcriptomes is not entirely without methodological justification.

On the other hand, we also acknowledge the reviewer's opinion that the interpretation of results in the context of whole blood should be approached with greater caution. Compared to PBMC or more purified immune cell samples, whole blood has a more complex cellular composition, with a higher proportion of cells such as neutrophils, and there may be some overlap in transcriptional signals among different cell subsets, which could affect the accuracy of distinguishing between certain similar cell populations or low-abundance cell subsets(PMID: 30726743). Therefore, we agree that the immune infiltration analysis in this study is more suitable as an auxiliary immune characterization and applicable for observing the overall trend of sepsis-related immune changes, rather than being overinterpreted as a precise quantification of the absolute abundance of each cell subpopulation.

In addition, multiple bioinformatics studies based on sepsis peripheral blood or whole blood transcriptomes have employed CIBERSORT to assess immune cell composition and its correlation with key genes, using it as an auxiliary analytical tool to reveal disease-related immune characteristics (PMID: 36817458, PMID: 37564869, PMID: 40140612). This indicates that in sepsis transcriptome research, the use of CIBERSORT for immune deconvolution of blood-based data has a certain practical application foundation. Based on the overall design of this study, our primary purpose in adopting this method is also to assist in evaluating the association between key genes and changes in the immune microenvironment, rather than using immune infiltration analysis as the sole evidence supporting the core conclusions of the entire paper.

Based on your suggestions, we will make further additions and adjustments to this section in the revised draft. First, in the methods section, we will supplement the rationale for using CIBERSORT and its application background in blood-based transcriptome analysis. Second, in the discussion section, we will explicitly add an explanation of the limitations of immune deconvolution in whole blood samples, emphasizing that the results should be interpreted with caution, especially for estimates of closely related cell populations and low-abundance cell populations. At the same time, we will also appropriately tone down related statements to avoid overextending the interpretation of these results. Through these revisions, we aim to make the positioning of this analysis more accurate and the presentation of results more cautious. Specific modifications can be found on Page 12-13, Line 252-260 Page 45, Line 659-668 and Page 47, Line 694-701 in revised manuscript with track changes. Thank you again for your valuable suggestions.

We are grateful for the invaluable feedback provided by the reviewers, and we look forward to the opportunity to improve our manuscript in response to their guidance.
