# Supplementary material for: Bioinformatics analysis reveals the association of bile acid metabolism-related genes with sepsis
Source: PLoS One. 2026 Jul 10;21(7):e0352431. doi: 10.1371/journal.pone.0352431 (PMC13354078; doi:10.1371/journal.pone.0352431)
Supplement: Peer review history

# Peer review: Decision Letter 0

**DOI:** 10.1371/journal.pone.0352431.r001

**Authors:** Suyan Tian

19 Nov 2025

PONE-D-25-38188

Bioinformatics analysis reveals the association of bile acid metabolism-related genes with sepsis

PLOS ONE

Dear Dr. Zhou,

Thank you for submitting your manuscript to PLOS ONE. After careful consideration, we feel that it has merit but does not fully meet PLOS ONE’s publication criteria as it currently stands. Therefore, we invite you to submit a revised version of the manuscript that addresses the points raised during the review process.

==============================

The authors need to address the following points before being considered for publication:

1. When identifying the differentially expressed genes (DEGs), why used p-values without any adjustment for multiple testing and a threshold of 0 for |logFC|?
2. For the training set, why combined the two datasets with small sample sizes. Plus, the authors barely mentioned to how to pre-process the gene expression data (if they directly downloaded the expression matrix from the GEO database, the used pre-process procedures may differ, which would significantly affect the results. Also, please give more details on how to deal with batch effect when combining the datasets.
3. No experimental validation has been conducted, please perform such a validation.
4. The bioinformatics methods used are very conventional, please justify in each step the conventional methods instead of more novel methods were used.
5. Please a more detailed discussion on the strengths and limitations of current study, particularly the clinical significance of constructing such a gene signature.

==============================

Please submit your revised manuscript by Jan 03 2026 11:59PM. If you will need more time than this to complete your revisions, please reply to this message or contact the journal office at plosone@plos.org. When you're ready to submit your revision, log on to https://www.editorialmanager.com/pone/ and select the 'Submissions Needing Revision' folder to locate your manuscript file.

Please include the following items when submitting your revised manuscript:

- A rebuttal letter that responds to each point raised by the academic editor and reviewer(s). You should upload this letter as a separate file labeled 'Response to Reviewers'.
- A marked-up copy of your manuscript that highlights changes made to the original version. You should upload this as a separate file labeled 'Revised Manuscript with Track Changes'.
- An unmarked version of your revised paper without tracked changes. You should upload this as a separate file labeled 'Manuscript'.

If you would like to make changes to your financial disclosure, please include your updated statement in your cover letter. Guidelines for resubmitting your figure files are available below the reviewer comments at the end of this letter.

If applicable, we recommend that you deposit your laboratory protocols in protocols.io to enhance the reproducibility of your results. Protocols.io assigns your protocol its own identifier (DOI) so that it can be cited independently in the future. For instructions see: https://journals.plos.org/plosone/s/submission-guidelines#loc-laboratory-protocols. Additionally, PLOS ONE offers an option for publishing peer-reviewed Lab Protocol articles, which describe protocols hosted on protocols.io. Read more information on sharing protocols at https://plos.org/protocols?utm_medium=editorial-email&utm_source=authorletters&utm_campaign=protocols.

We look forward to receiving your revised manuscript.

Kind regards,

Suyan Tian

Academic Editor

PLOS ONE

**Journal Requirements:**

1. When submitting your revision, we need you to address these additional requirements.

Please ensure that your manuscript meets PLOS ONE's style requirements, including those for file naming. The PLOS ONE style templates can be found at

https://journals.plos.org/plosone/s/file?id=wjVg/PLOSOne_formatting_sample_main_body.pdf and

https://journals.plos.org/plosone/s/file?id=ba62/PLOSOne_formatting_sample_title_authors_affiliations.pdf

2. Thank you for stating the following financial disclosure:

This study is sponsored by the National Natural Science Foundation of China (General Program No.82171738) and the Special Funds for Health Science and Technology Development of Nanjing City (No. YKK20145).

Please state what role the funders took in the study.  If the funders had no role, please state: "The funders had no role in study design, data collection and analysis, decision to publish, or preparation of the manuscript."

If this statement is not correct you must amend it as needed.

Please include this amended Role of Funder statement in your cover letter; we will change the online submission form on your behalf.

3. We are unable to open your Supporting Information file “Table S3-string_interactions_short.tsv” . Please kindly revise as necessary and re-upload.

4. If the reviewer comments include a recommendation to cite specific previously published works, please review and evaluate these publications to determine whether they are relevant and should be cited. There is no requirement to cite these works unless the editor has indicated otherwise.

[Note: HTML markup is below. Please do not edit.]

Reviewers' comments:

Reviewer's Responses to Questions

**Comments to the Author**

1. Is the manuscript technically sound, and do the data support the conclusions?

The manuscript must describe a technically sound piece of scientific research with data that supports the conclusions. Experiments must have been conducted rigorously, with appropriate controls, replication, and sample sizes. The conclusions must be drawn appropriately based on the data presented.

Reviewer #1: Yes

**********

2. Has the statistical analysis been performed appropriately and rigorously?

Reviewer #1: Yes

**********

3. Have the authors made all data underlying the findings in their manuscript fully available?

The PLOS Data policy requires authors to make all data underlying the findings described in their manuscript fully available without restriction, with rare exception (please refer to the Data Availability Statement in the manuscript PDF file). The data should be provided as part of the manuscript or its supporting information, or deposited to a public repository. For example, in addition to summary statistics, the data points behind means, medians and variance measures should be available. If there are restrictions on publicly sharing data—e.g. participant privacy or use of data from a third party—those must be specified.

Reviewer #1: Yes

**********

4. Is the manuscript presented in an intelligible fashion and written in standard English?

PLOS ONE does not copyedit accepted manuscripts, so the language in submitted articles must be clear, correct, and unambiguous. Any typographical or grammatical errors should be corrected at revision, so please note any specific errors here.

Reviewer #1: No

**********

5. Review Comments to the Author

Please use the space provided to explain your answers to the questions above. You may also include additional comments for the author, including concerns about dual publication, research ethics, or publication ethics. (Please upload your review as an attachment if it exceeds 20,000 characters)

**Reviewer #1:**  1.The article contains some grammatical errors and is overly verbose; it is recommended to simplify and polish the text.

2.Is the threshold |logFC| > 0 in section 2.2 overly lenient? Please briefly explain the criteria and rationale for this setting.

3.It is recommended to incorporate additional clinical validation experiments to enhance the accuracy and reliability of the analytical results.

**********

6. PLOS authors have the option to publish the peer review history of their article (what does this mean?). If published, this will include your full peer review and any attached files.

If you choose “no”, your identity will remain anonymous but your review may still be made public.

**Do you want your identity to be public for this peer review?**  For information about this choice, including consent withdrawal, please see our Privacy Policy.

Reviewer #1: No

**********

[NOTE: If reviewer comments were submitted as an attachment file, they will be attached to this email and accessible via the submission site. Please log into your account, locate the manuscript record, and check for the action link "View Attachments". If this link does not appear, there are no attachment files.]

To ensure your figures meet our technical requirements, please review our figure guidelines: https://journals.plos.org/plosone/s/figures

You may also use PLOS’s free figure tool, NAAS, to help you prepare publication quality figures: https://journals.plos.org/plosone/s/figures#loc-tools-for-figure-preparation.

NAAS will assess whether your figures meet our technical requirements by comparing each figure against our figure specifications.

# Author comment: Author response to Decision Letter 1

**DOI:** 10.1371/journal.pone.0352431.r002

6 Jan 2026

Dear Editors and Reviewers:

We thank the reviewers for reviewing the manuscript and for their constructive comments during the review process. Your comments, along with those of the other reviewers, are extremely important to our article. We greatly appreciate your insights, which have significantly contributed to enhancing the quality of our work. In this revision, we have prepared a detailed point-by-point response letter for you and the other valued reviewers. This letter acknowledges your feedback and clearly outlines the revisions we have made. In response to the reviewers' comments, we have made extensive revisions to the manuscript. If there are any further modifications you would like us to consider, we would be more than willing to accommodate them, and we genuinely appreciate your assistance. [PLOS One] is a highly esteemed journal, and we hope that our manuscript can be considered for publication. Thank you very much for your support. We would like to express our sincere gratitude to you and the reviewers for your valuable comments on our manuscript. In light of the thorough and insightful feedback provided, we have revised the manuscript accordingly. We have added relevant content to address the reviewers' suggestions, and we have rewritten several sections to ensure clarity and completeness. We hope that these modifications meet the reviewers' expectations and enhance the overall quality of the manuscript.

The authors need to address the following points before being considered for publication. Responds to the reviewer’s comments:

1.When identifying the differentially expressed genes (DEGs), why used p-values without any adjustment for multiple testing and a threshold of 0 for |logFC|?

Reply to the reviewers: Thank you for carefully reviewing our manuscript and providing valuable comments. When we initially screened differentially expressed genes (DEGs), the threshold was set as |logFC| > 0 and p < 0.05, mainly for the following considerations: Firstly, the aim of this study is to comprehensively explore as many candidate genes related to bile acid metabolism associated with sepsis as possible, in order to provide a sufficient gene set foundation for subsequent protein interaction networks (PPIs), enrichment analysis, and machine learning model construction. If overly strict thresholds (such as high |logFC| or multiple corrected p values) are adopted in the initial screening stage, it may lead to the omission of some genes with potential biological significance but relatively small expression variations, thereby affecting the integrity of the overall network analysis. In the subsequent analysis, we gradually narrowed down the candidate scope through multi-level validation (including CytoHubba algorithm screening, LASSO regression, SVM-RFE cross-validation) to ensure the robustness and reliability of the final key genes from statistical and biological perspectives. In addition, similar initial screening criteria have been used in genome-wide exploratory study to ensure broad coverage of the analysis (PMID: 39021997). In this article, although a threshold of |logFC| > 1 was used in the preliminary analysis, the threshold was adjusted to |logFC| > 0 and p < 0.05 when identifying ARDEGs, indicating that the threshold selection for fold-change in differential analysis can be appropriately broadened so as to incorporate as many DEGs as possible. We fully agree with the reviewers' suggestions, in future further research, multiple hypothesis testing corrections (such as FDR) and a more reasonable threshold for logFC will be considered in the initial screening phase to enhance the rigor of the screening criteria and the persuasiveness of the results. Thank you again for your valuable suggestions.

2.For the training set, why combined the two datasets with small sample sizes. Plus, the authors barely mentioned to how to pre-process the gene expression data (if they directly downloaded the expression matrix from the GEO database, the used pre-process procedures may differ, which would significantly affect the results. Also, please give more details on how to deal with batch effect when combining the datasets.

Reply to the reviewers: Thank you for carefully reviewing our manuscript and providing valuable comments. In this study, both the GSE28750 and GSE95233 are derived from the same platform (GPL570) and have the same organizational source.To ensure the statistical performance and the robustness of the model, the two are combined as a training set. In terms of data preprocessing, firstly, the probe numbers were converted into gene symbols according to the annotation file of the corresponding platform of each dataset.For datasets from different platforms, after probe conversion only the intersecting genes were retained to ensure data consistency. Subsequently, the normalizeBetweenArrays function in the R package limma was used for standardization processing to eliminate systematic errors between samples. Finally, the batch effect correction was done using the ComBat function in the R-package sva, and the correction effect was verified by principal component analysis (PCA), and the results showed that the batch difference was effectively eliminated. We have further supplemented the unified preprocessing process description for the original data in the revised draft, so that readers can have a clearer understanding of the data integration and correction process.For specific details of the modifications, please refer to page 4-5 and line 85-100.

Thank you again for your valuable suggestions.

3. No experimental validation has been conducted, please perform such a validation.

Reply to the reviewers: Thank you for carefully reviewing our manuscript and providing valuable comments. We fully understand your suggestions regarding experimental verification. We have added qRT-PCR experiments in the revised draft to validate the expression changes of key genes in sepsis samples. Specifically, we examined the relative expression levels of five key genes (ABCC2�AGXT�PEX2�PECR�EPHX2) in the sepsis group and the control group, and the results showed that the transcriptional expression levels of ABCC2 and AGXT in the sepsis group were significantly higher than that in the control group, and there were no statistically significant differences between the two groups in PEX2, PECR and EPHX2. The reliability and biological relevance of the key genes were further verified at the experimental level and results are generally consistent with the bioinformatics analysis results. We have supplemented the qRT-PCR experimental data in the results section of the revised draft, and expounded on its consistency with the bioinformatics analysis results and its biological significance in the discussion section. Thank you again for your valuable suggestions.

4.The bioinformatics methods used are very conventional, please justify in each step the conventional methods instead of more novel methods were used.

Reply to the reviewers: Thank you for carefully reviewing our manuscript and providing valuable comments. The major strength of bioinformatics methods adopted in this study pertains to its robustness, interpretability and reproducibility. In response to the questions you raised, we have explained the scientific basis and suitability of selecting traditional algorithms for each step in the revised draft. Specifically, the data in this study is derived from multi-platform chip data. Classical algorithm “sva” and “limma” have mature theoretical foundations and widely verified reliability in such data processing. The STRING database and the cytoHubba plugin were applied to PPI network analysis to ensure the stability and comparability of the results. GO/KEGG and GSEA are internationally recognized functional annotation systems, suitable for systematic analysis of disease mechanisms. Logistic Regression, SVM-RFE and LASSO models have good generalization and biological explanatory power in the analysis of small sample high-dimensional data. The CIBERSORT algorithm has been widely verified in immune infiltration analysis in blood samples and can accurately reflect the composition of immune cells. Given the biological significance and clinical translational potential of the results focused on in this study, we believe that adopting conventional bioinformatics methods can better ensure the stability and repeatability of the analysis. In subsequent research, we will further explore the application potential of emerging algorithms such as deep learning to expand the depth and breadth of the analysis.

For specific details of the modifications, please refer to methodology section.

Thank you again for your valuable suggestions.

5. Please a more detailed discussion on the strengths and limitations of current study, particularly the clinical significance of constructing such a gene signature.

Reply to the reviewers: Thank you for carefully reviewing our manuscript and providing valuable comments. We have provided a more systematic elaboration on the advantages and limitations of the research as well as the clinical significance of the constructed gene characteristics. In the revised draft, we have further emphasized multiple advantages of this study, including multi-dataset integration�batch effect control and multi-algorithm joint screening for key genes (SVM-RFE algorithm and LASSO regression), as well as the evaluation of model performance through internal and external validation. Meanwhile, we have supplemented the objective limitations of this study in terms of data source�sample size�lack of experimental validation and clinical translation, and further clarified the application prospects of this model in early diagnosis of sepsis, individualized risk assessment and discovery of potential therapeutic targets. For specific details of the modifications, please refer to page 47-48 and line 654-681.Thank you again for your valuable suggestions.

Journal Requirements:

1. When submitting your revision, we need you to address these additional requirements.

Please ensure that your manuscript meets PLOS ONE's style requirements, including those for file naming. The PLOS ONE style templates can be found at

https://journals.plos.org/plosone/s/file?id=wjVg/PLOSOne_formatting_sample_main_body.pdf and

https://journals.plos.org/plosone/s/file?id=ba62/PLOSOne_formatting_sample_title_authors_affiliations.pdf

Reply to the editors: Thank you for carefully reviewing our manuscript and providing valuable comments. In strict accordance with the requirements of the PLOS ONE official template, we have comprehensively adjusted the title page, abstract, body level, chart naming and file naming format in the new revised draft, making the manuscript fully complied with the journal specifications in terms of structure and layout. Thank you again for your valuable suggestions.

2. Thank you for stating the following financial disclosure:

This study is sponsored by the National Natural Science Foundation of China (General Program No.82171738) and the Special Funds for Health Science and Technology Development of Nanjing City (No. YKK20145). Please state what role the funders took in the study. If the funders had no role, please state: "The funders had no role in study design, data collection and analysis, decision to publish, or preparation of the manuscript." If this statement is not correct you must amend it as needed. Please include this amended Role of Funder statement in your cover letter; we will change the online submission form on your behalf.

Reply to the editor: Thank you for carefully reviewing our manuscript and providing valuable comments. The author(s) received no specific funding for this work, the previously incorrect expressions have been corrected. We have updated the relevant instructions in the manuscript. Thank you again for your valuable suggestions.

3.We are unable to open your Supporting Information file“Table S3-string_interactions_short.tsv”. Please kindly revise as necessary and re-upload.

Reply to the editors: Thank you for carefully reviewing our manuscript and providing valuable comments. Regarding the issue you mentioned that the “Supporting Information” file cannot be opened, we have re-exported and uploaded the file in the new revised draft, uniformly using the XLS format to ensure that it can be opened normally in common systems and software environments. Thank you again for your valuable suggestions.

4.If the reviewer comments include a recommendation to cite specific previously published works, please review and evaluate these publications to determine whether they are relevant and should be cited. There is no requirement to cite these works unless the editor has indicated otherwise.

Reply to the editors: Thank you very much for reminding us. Such scenario was not encountered in the current study. Thank you again for your valuable suggestions.

Reviewers' comments:

Reviewer's Responses to Questions

Comments to the Author

1. Is the manuscript technically sound, and do the data support the conclusions?

The manuscript must describe a technically sound piece of scientific research with data that supports the conclusions. Experiments must have been conducted rigorously, with appropriate controls, replication, and sample sizes. The conclusions must be drawn appropriately based on the data presented.

Reply to the reviewers: Thank you for carefully reviewing our manuscript and providing valuable comments. We have conducted a comprehensive assessment of the recommended literature based on your suggestions and selected the best ones for citation in combination with the relevance of the research topic, methods and content, in order to further improve the theoretical and methodological support of the manuscript. For literature with significant differences in research directions or methods, we do not include them in the scope of citation to maintain the concentration and logical consistency of the discussion. Thank you again for your valuable suggestions.

Reviewer #1: Yes

2. Has the statistical analysis been performed appropriately and rigorously?

Reviewer #1: Yes

3. Have the authors made all data underlying the findings in their manuscript fully available?

The PLOS Data policy requires authors to make all data underlying the findings described in their manuscript fully available without restriction, with rare exception (please refer to the Data Availability Statement in the manuscript PDF file). The data should be provided as part of the manuscript or its supporting information, or deposited to a public repository. For example, in addition to summary statistics, the data points behind means, medians and variance measures should be available. If there are restrictions on publicly sharing data—e.g. participant privacy or use of data from a third party—those must be specified.

Reviewer #1: Yes

4. Is the manuscript presented in an intelligible fashion and written in standard English?

PLOS ONE does not copyedit accepted manuscripts, so the language in submitted articles must be clear, correct, and unambiguous. Any typographical or grammatical errors should be corrected at revision, so please note any specific errors here.

Reviewer #1: No

5. Review Comments to the Author

Please use the space provided to explain your answers to the questions above. You may also include additional comments for the author, including concerns about dual publication, research ethics, or publication ethics. (Please upload your review as an attachment if it exceeds 20,000 characters)

Reviewer #1: 1.The article contains some grammatical errors and is overly verbose; it is recommended to simplify and polish the text.

Reply to the reviewers: Thank you for carefully reviewing our manuscript and providing valuable comments. We have entrusted a professional proofreading agency to conduct a comprehensive language editing for the manuscript to ensure that the language expression is accurate and luent, and meets academic standards. Thank you again for your valuable suggestions.

2.Is the threshold |logFC| > 0 in section 2.2 overly lenient? Please briefly explain the criteria and rationale for this setting.

Reply to the reviewers: Thank you for carefully reviewing ou

Attachment Submitted filename: Response to Reviewers.doc

# Peer review: Decision Letter 1

**DOI:** 10.1371/journal.pone.0352431.r003

**Authors:** Suyan Tian, Suyan Tian

23 Apr 2026

PONE-D-25-38188R1

Bioinformatics analysis reveals the association of bile acid metabolism-related genes with sepsis

PLOS One

Dear Dr. Zhou,

Thank you for submitting your manuscript to PLOS ONE. After careful consideration, we feel that it has merit but does not fully meet PLOS ONE’s publication criteria as it currently stands. Therefore, we invite you to submit a revised version of the manuscript that addresses the points raised during the review process.

Please address the additional points raised by Reviewer 2.

Please submit your revised manuscript by Jun 07 2026 11:59PM. If you will need more time than this to complete your revisions, please reply to this message or contact the journal office at plosone@plos.org. When you're ready to submit your revision, log on to https://www.editorialmanager.com/pone/ and select the 'Submissions Needing Revision' folder to locate your manuscript file.

Please include the following items when submitting your revised manuscript:

- A letter that responds to each point raised by the academic editor and reviewer(s). You should upload this letter as a separate file labeled 'Response to Reviewers'.
- A marked-up copy of your manuscript that highlights changes made to the original version. You should upload this as a separate file labeled 'Revised Manuscript with Track Changes'.
- An unmarked version of your revised paper without tracked changes. You should upload this as a separate file labeled 'Manuscript'.

If you would like to make changes to your financial disclosure, please include your updated statement in your cover letter. Guidelines for resubmitting your figure files are available below the reviewer comments at the end of this letter.

If applicable, we recommend that you deposit your laboratory protocols in protocols.io to enhance the reproducibility of your results. Protocols.io assigns your protocol its own identifier (DOI) so that it can be cited independently in the future. For instructions see: https://journals.plos.org/plosone/s/submission-guidelines#loc-laboratory-protocols. Additionally, PLOS ONE offers an option for publishing peer-reviewed Lab Protocol articles, which describe protocols hosted on protocols.io. Read more information on sharing protocols at https://plos.org/protocols?utm_medium=editorial-email&utm_source=authorletters&utm_campaign=protocols.

As the corresponding author, your ORCID iD is verified in the submission system and will appear in the published article. PLOS supports the use of ORCID, and we encourage all coauthors to register for an ORCID iD and use it as well. Please encourage your coauthors to verify their ORCID iD within the submission system before final acceptance, as unverified ORCID iDs will not appear in the published article. *Only*  the individual author can complete the verification step; PLOS staff *cannot*  verify ORCID iDs on behalf of authors.

We look forward to receiving your revised manuscript.

Kind regards,

Suyan Tian

Academic Editor

PLOS One

Journal Requirements:

If the reviewer comments include a recommendation to cite specific previously published works, please review and evaluate these publications to determine whether they are relevant and should be cited. There is no requirement to cite these works unless the editor has indicated otherwise.

Please review your reference list to ensure that it is complete and correct. If you have cited papers that have been retracted, please include the rationale for doing so in the manuscript text, or remove these references and replace them with relevant current references. Any changes to the reference list should be mentioned in the rebuttal letter that accompanies your revised manuscript. If you need to cite a retracted article, indicate the article’s retracted status in the References list and also include a citation and full reference for the retraction notice.

[Note: HTML markup is below. Please do not edit.]

Reviewers' comments:

Reviewer's Responses to Questions

**Comments to the Author**

1. If the authors have adequately addressed your comments raised in a previous round of review and you feel that this manuscript is now acceptable for publication, you may indicate that here to bypass the “Comments to the Author” section, enter your conflict of interest statement in the “Confidential to Editor” section, and submit your "Accept" recommendation.

Reviewer #2: All comments have been addressed

**********

2. Is the manuscript technically sound, and do the data support the conclusions?

The manuscript must describe a technically sound piece of scientific research with data that supports the conclusions. Experiments must have been conducted rigorously, with appropriate controls, replication, and sample sizes. The conclusions must be drawn appropriately based on the data presented.

Reviewer #2: Yes

**********

3. Has the statistical analysis been performed appropriately and rigorously?

Reviewer #2: Yes

**********

4. Have the authors made all data underlying the findings in their manuscript fully available?

The PLOS Data policy requires authors to make all data underlying the findings described in their manuscript fully available without restriction, with rare exception (please refer to the Data Availability Statement in the manuscript PDF file). The data should be provided as part of the manuscript or its supporting information, or deposited to a public repository. For example, in addition to summary statistics, the data points behind means, medians and variance measures should be available. If there are restrictions on publicly sharing data—e.g. participant privacy or use of data from a third party—those must be specified.

Reviewer #2: Yes

**********

5. Is the manuscript presented in an intelligible fashion and written in standard English?

PLOS ONE does not copyedit accepted manuscripts, so the language in submitted articles must be clear, correct, and unambiguous. Any typographical or grammatical errors should be corrected at revision, so please note any specific errors here.

Reviewer #2: Yes

**********

6. Review Comments to the Author

Please use the space provided to explain your answers to the questions above. You may also include additional comments for the author, including concerns about dual publication, research ethics, or publication ethics. (Please upload your review as an attachment if it exceeds 20,000 characters)

Reviewer #2: This study attempts to identify bile acid metabolism-related diagnostic markers for sepsis using a standard bioinformatics pipeline followed by qRT-PCR validation. While the topic has some interest, I have three major concerns regarding methodology, analysis appropriateness, and validation completeness. A major revision is required before the manuscript can be considered for publication.

1. The authors used |logFC| > 0 and raw p < 0.05 without multiple testing correction to identify DEGs, claiming an “exploratory” approach to avoid missing biologically relevant genes with small effect sizes. This rationale is insufficient. No evidence is provided that genes included only under this loose threshold actually contribute to the final diagnostic model or improve its performance. To justify this choice, the authors must perform a sensitivity analysis comparing the current loose threshold with a more rigorous one (e.g., |logFC| > 0.5, FDR < 0.05). The comparison should include at least: (a) the number of DEGs and BAMRDEGs identified; (b) the hub genes selected; (c) the key genes selected by LASSO+SVM-RFE; and (d) the diagnostic AUC in both training and validation datasets. If the results are similar, the loose threshold may be acceptable; if not, the stricter criteria must be adopted. This analysis should be presented as a supplementary table or figure.

2. The authors used CIBERSORT to estimate immune cell infiltration. CIBERSORT was originally developed and validated for tumor tissue transcriptomes, where cell type composition is markedly different from peripheral blood. Its application to whole blood samples is not standard and may produce unreliable estimates, especially for rare or closely related cell populations. The authors should either: (a) provide validation or cite studies that have successfully applied CIBERSORT to blood samples with similar immune cell deconvolution goals; or (b) use an alternative method specifically designed for blood, such as ImmuCellAI, xCell, or a reference-based deconvolution approach with a blood-derived signature matrix. Alternatively, the authors could remove the immune infiltration analysis if it does not directly support the main conclusion.

**********

7. PLOS authors have the option to publish the peer review history of their article (what does this mean?). If published, this will include your full peer review and any attached files.

If you choose “no”, your identity will remain anonymous but your review may still be made public.

**Do you want your identity to be public for this peer review?**  For information about this choice, including consent withdrawal, please see our Privacy Policy.

Reviewer #2: No

**********

[NOTE: If reviewer comments were submitted as an attachment file, they will be attached to this email and accessible via the submission site. Please log into your account, locate the manuscript record, and check for the action link "View Attachments". If this link does not appear, there are no attachment files.]

To ensure your figures meet our technical requirements, please review our figure guidelines: https://journals.plos.org/plosone/s/figures

You may also use PLOS’s free figure tool, NAAS, to help you prepare publication quality figures: https://journals.plos.org/plosone/s/figures#loc-tools-for-figure-preparation.

NAAS will assess whether your figures meet our technical requirements by comparing each figure against our figure specifications.

# Author comment: Author response to Decision Letter 2

**DOI:** 10.1371/journal.pone.0352431.r004

27 May 2026

Reviewer #2: This study attempts to identify bile acid metabolism-related diagnostic markers for sepsis using a standard bioinformatics pipeline followed by qRT-PCR validation. While the topic has some interest, I have three major concerns regarding methodology, analysis appropriateness, and validation completeness. A major revision is required before the manuscript can be considered for publication.

1. The authors used |logFC| > 0 and raw p < 0.05 without multiple testing correction to identify DEGs, claiming an “exploratory” approach to avoid missing biologically relevant genes with small effect sizes. This rationale is insufficient. No evidence is provided that genes included only under this loose threshold actually contribute to the final diagnostic model or improve its performance. To justify this choice, the authors must perform a sensitivity analysis comparing the current loose threshold with a more rigorous one (e.g., |logFC| > 0.5, FDR < 0.05). The comparison should include at least: (a) the number of DEGs and BAMRDEGs identified; (b) the hub genes selected; (c) the key genes selected by LASSO+SVM-RFE; and (d) the diagnostic AUC in both training and validation datasets. If the results are similar, the loose threshold may be acceptable; if not, the stricter criteria must be adopted. This analysis should be presented as a supplementary table or figure.

Reply to the reviewers: Thank you for carefully reviewing our manuscript and providing valuable comments. We fully understand your concerns regarding the threshold settings for the initial screening of DEGs. Regarding the use of |logFC| > 0 and raw P < 0.05 as the preliminary criteria for DEGs in this study, we would like to further clarify that this step was primarily employed for exploratory screening of candidate genes, rather than serving as the sole basis for final key genes identification or diagnostic model construction. Since the peripheral blood transcriptome data included in this study were derived from different independent cohorts, and sepsis exhibits strong clinical and biological heterogeneity, some biological changes related to bile acid metabolism are more likely to manifest as expression changes with small effect sizes but stable directionality. In this research context, if a high fold change threshold is applied at the initial screening stage, it may prematurely exclude some candidate genes with potential biological significance.

More importantly, the final results of this study are not directly based on the DEGs screened under all lenient thresholds. Instead, after the initial screening, further multi-round contraction analyses were conducted, including intersection with bile acid metabolism-related gene sets, PPI network screening, intersection analysis of five algorithms from cytoHubba, Logistic regression, SVM-RFE, and LASSO regression. In other words, the primary purpose of the initial DEG screening is to enhance the sensitivity in the candidate signal discovery phase, while the final key genes and diagnostic model are established based on continuous screening, cross-optimization and independent validation. Therefore, we believe that the current threshold is more suitable as a pre-screening criterion for the candidate gene discovery phase in this study, rather than the sole criterion for directly determining the final conclusions.

Furthermore, this study did not stop at the level of simple differential analysis, instead, further validated the diagnostic performance of the model in the dataset GSE13904, and experimentally verified the expression trends of key genes using qRT-PCR. This analytical framework of "exploratory initial screening + multi-round feature constriction + external validation + experimental validation" reduces the impact of threshold settings in individual steps on the stability of the final results to a certain extent. Similarly, some bioinformatics studies have also adopted a screening strategy of |logFC| > 0 and P < 0.05 during the candidate genes exploration stage, followed by further screening of key molecules through network analysis or machine learning methods (PMID: 34955918, PMID: 39916852, PMID: 40750912). Thank you again for your valuable suggestions.

2. The authors used CIBERSORT to estimate immune cell infiltration. CIBERSORT was originally developed and validated for tumor tissue transcriptomes, where cell type composition is markedly different from peripheral blood. Its application to whole blood samples is not standard and may produce unreliable estimates, especially for rare or closely related cell populations. The authors should either: (a) provide validation or cite studies that have successfully applied CIBERSORT to blood samples with similar immune cell deconvolution goals; or (b) use an alternative method specifically designed for blood, such as ImmuCellAI, xCell, or a reference-based deconvolution approach with a blood-derived signature matrix. Alternatively, the authors could remove the immune infiltration analysis if it does not directly support the main conclusion.

Reply to the reviewers: Thank you for carefully reviewing our manuscript and providing valuable comments. We greatly appreciate your attention to the applicability of immune infiltration analysis methods. Regarding the use of CIBERSORT for estimating immune cell composition in the peripheral blood transcriptome in this study, we have carefully re-examined the relevant methodological basis. We understand your concern that this method may have limitations in the whole blood samples, which is crucial for the rigor of result interpretation.

Meanwhile, we would like to provide an appropriate explanation regarding this issue. CIBERSORT is not an immune infiltration tool developed exclusively for tumor tissues. Its original method is based on the LM22 leukocyte signature matrix, primarily used to estimate the relative abundance of 22 types of human immune cell subsets in complex mixed samples, the establishment of methodology itself includes the modeling and validation of the expression characteristics related to leukocytes(PMID: 25822800). Therefore, from a methodological perspective, simply interpreting it as a tool "only applicable to tumor tissues" is not entirely accurate. Based on this, we believe that using CIBERSORT for preliminary immune deconvolution analysis in peripheral blood transcriptomes is not entirely without methodological justification.

On the other hand, we also acknowledge the reviewer's opinion that the interpretation of results in the context of whole blood should be approached with greater caution. Compared to PBMC or more purified immune cell samples, whole blood has a more complex cellular composition, with a higher proportion of cells such as neutrophils, and there may be some overlap in transcriptional signals among different cell subsets, which could affect the accuracy of distinguishing between certain similar cell populations or low-abundance cell subsets(PMID: 30726743). Therefore, we agree that the immune infiltration analysis in this study is more suitable as an auxiliary immune characterization and applicable for observing the overall trend of sepsis-related immune changes, rather than being overinterpreted as a precise quantification of the absolute abundance of each cell subpopulation.

In addition, multiple bioinformatics studies based on sepsis peripheral blood or whole blood transcriptomes have employed CIBERSORT to assess immune cell composition and its correlation with key genes, using it as an auxiliary analytical tool to reveal disease-related immune characteristics (PMID: 36817458, PMID: 37564869, PMID: 40140612). This indicates that in sepsis transcriptome research, the use of CIBERSORT for immune deconvolution of blood-based data has a certain practical application foundation. Based on the overall design of this study, our primary purpose in adopting this method is also to assist in evaluating the association between key genes and changes in the immune microenvironment, rather than using immune infiltration analysis as the sole evidence supporting the core conclusions of the entire paper.

Based on your suggestions, we will make further additions and adjustments to this section in the revised draft. First, in the methods section, we will supplement the rationale for using CIBERSORT and its application background in blood-based transcriptome analysis. Second, in the discussion section, we will explicitly add an explanation of the limitations of immune deconvolution in whole blood samples, emphasizing that the results should be interpreted with caution, especially for estimates of closely related cell populations and low-abundance cell populations. At the same time, we will also appropriately tone down related statements to avoid overextending the interpretation of these results. Through these revisions, we aim to make the positioning of this analysis more accurate and the presentation of results more cautious. Specific modifications can be found on Page 12-13, Line 252-260 Page 45, Line 659-668 and Page 47, Line 694-701 in revised manuscript with track changes. Thank you again for your valuable suggestions.

Attachment Submitted filename: Response to Reviewers 5.27.doc

# Peer review: Decision Letter 2

**DOI:** 10.1371/journal.pone.0352431.r005

**Authors:** Suyan Tian, Suyan Tian, Suyan Tian

11 Jun 2026

<p>Bioinformatics analysis reveals the association of bile acid metabolism-related genes with sepsis

PONE-D-25-38188R2

Dear Dr. Zhou,

We’re pleased to inform you that your manuscript has been judged scientifically suitable for publication and will be formally accepted for publication once it meets all outstanding technical requirements.

Within one week, you’ll receive an e-mail detailing the required amendments. When these have been addressed, you’ll receive a formal acceptance letter and your manuscript will be scheduled for publication.

An invoice will be generated when your article is formally accepted. Please note, if your institution has a publishing partnership with PLOS and your article meets the relevant criteria, all or part of your publication costs will be covered. Please make sure your user information is up-to-date by logging into Editorial Manager at Editorial Manager® and clicking the ‘Update My Information' link at the top of the page. For questions related to billing, please contact billing support.

If your institution or institutions have a press office, please notify them about your upcoming paper to help maximize its impact. If they’ll be preparing press materials, please inform our press team as soon as possible -- no later than 48 hours after receiving the formal acceptance. Your manuscript will remain under strict press embargo until 2 pm Eastern Time on the date of publication. For more information, please contact onepress@plos.org.

Kind regards,

Suyan Tian

Academic Editor

PLOS One

Additional Editor Comments (optional):

Reviewers' comments:

Reviewer's Responses to Questions

**Comments to the Author**

1. If the authors have adequately addressed your comments raised in a previous round of review and you feel that this manuscript is now acceptable for publication, you may indicate that here to bypass the “Comments to the Author” section, enter your conflict of interest statement in the “Confidential to Editor” section, and submit your "Accept" recommendation.

Reviewer #2: All comments have been addressed

**********

2. Is the manuscript technically sound, and do the data support the conclusions?

The manuscript must describe a technically sound piece of scientific research with data that supports the conclusions. Experiments must have been conducted rigorously, with appropriate controls, replication, and sample sizes. The conclusions must be drawn appropriately based on the data presented.

Reviewer #2: Yes

**********

3. Has the statistical analysis been performed appropriately and rigorously?

Reviewer #2: Yes

**********

4. Have the authors made all data underlying the findings in their manuscript fully available?

The PLOS Data policy requires authors to make all data underlying the findings described in their manuscript fully available without restriction, with rare exception (please refer to the Data Availability Statement in the manuscript PDF file). The data should be provided as part of the manuscript or its supporting information, or deposited to a public repository. For example, in addition to summary statistics, the data points behind means, medians and variance measures should be available. If there are restrictions on publicly sharing data—e.g. participant privacy or use of data from a third party—those must be specified.

Reviewer #2: Yes

**********

5. Is the manuscript presented in an intelligible fashion and written in standard English?

PLOS ONE does not copyedit accepted manuscripts, so the language in submitted articles must be clear, correct, and unambiguous. Any typographical or grammatical errors should be corrected at revision, so please note any specific errors here.

Reviewer #2: Yes

**********

6. Review Comments to the Author

Please use the space provided to explain your answers to the questions above. You may also include additional comments for the author, including concerns about dual publication, research ethics, or publication ethics. (Please upload your review as an attachment if it exceeds 20,000 characters)

Reviewer #2: (No Response)

**********

7. PLOS authors have the option to publish the peer review history of their article (what does this mean?). If published, this will include your full peer review and any attached files.

If you choose “no”, your identity will remain anonymous but your review may still be made public.

**Do you want your identity to be public for this peer review?**  For information about this choice, including consent withdrawal, please see our Privacy Policy.

Reviewer #2: No

**********

# Editor report: Acceptance letter

**DOI:** 10.1371/journal.pone.0352431.r006

**Authors:** Suyan Tian, Suyan Tian, Suyan Tian

PONE-D-25-38188R2

PLOS One

Dear Dr. Zhou,

I'm pleased to inform you that your manuscript has been deemed suitable for publication in PLOS One. Congratulations! Your manuscript is now being handed over to our production team.

At this stage, our production department will prepare your paper for publication. This includes ensuring the following:

* All references, tables, and figures are properly cited

* All relevant supporting information is included in the manuscript submission,

* There are no issues that prevent the paper from being properly typeset

You will receive further instructions from the production team, including instructions on how to review your proof when it is ready. Please keep in mind that we are working through a large volume of accepted articles, so please give us a few days to review your paper and let you know the next and final steps.

Lastly, if your institution or institutions have a press office, please let them know about your upcoming paper now to help maximize its impact. If they'll be preparing press materials, please inform our press team within the next 48 hours. Your manuscript will remain under strict press embargo until 2 pm Eastern Time on the date of publication. For more information, please contact onepress@plos.org.

You will receive an invoice from PLOS for your publication fee after your manuscript has reached the completed accept phase. If you receive an email requesting payment before acceptance or for any other service, this may be a phishing scheme. Learn how to identify phishing emails and protect your accounts at https://explore.plos.org/phishing.

If we can help with anything else, please email us at customercare@plos.org.

Thank you for submitting your work to PLOS ONE and supporting open access.

Kind regards,

PLOS ONE Editorial Office Staff

on behalf of

Dr. Suyan Tian

Academic Editor

PLOS One
